# Elevation in lung volume and preventing catastrophic airway closure in asthmatics during bronchoconstriction

**DOI:** 10.1371/journal.pone.0208337

**Published:** 2018-12-19

**Authors:** Juan S. Osorio-Valencia, Chanikarn Wongviriyawong, Tilo Winkler, Vanessa J. Kelly, Robert S. Harris, Jose G. Venegas

**Affiliations:** 1 Department of Computer Science, Graduate Program in Biomedical Computing, Technical University of Munich, Munich, Germany; 2 Department of Anesthesia and Critical Care, Massachusetts General Hospital and Harvard Medical School, Boston, Massachusetts, United States of America; 3 Department of Mechanical Engineering, Massachusetts Institute of Technology, Cambridge, Massachusetts, United States of America; 4 Department of Medicine, Pulmonary and Critical Care Unit, Massachusetts General Hospital and Harvard Medical School, Boston, Massachusetts, United States of America; Karolinska Institutet, SWEDEN

## Abstract

**Background:**

Asthma exacerbations cause lung hyperinflation, elevation in load to inspiratory muscles, and decreased breathing capacity that, in severe cases, may lead to inspiratory muscle fatigue and respiratory failure. Hyperinflation has been attributed to a passive mechanical origin; a respiratory system time-constant too long for full exhalation. However, because the increase in volume is also concurrent with activation of inspiratory muscles during exhalation it is unclear whether hyperinflation in broncho-constriction is a passive phenomenon or is actively controlled to avoid airway closure.

**Methods:**

Using CT scanning, we measured the distensibility of individual segmental airways relative to that of their surrounding parenchyma in seven subjects with asthma and nine healthy controls. With this data we tested whether the elevation of lung volume measured after methacholine (MCh) provocation was associated with airway narrowing, or to the volume required to preventing airway closure. We also tested whether the reduction in FVC post-MCh could be attributed to gas trapped behind closed segmental airways.

**Findings:**

The changes in lung volume by MCh in subjects with and without asthma were inversely associated with their reduction in average airway lumen. This finding would be inconsistent with hyperinflation by passive elevation of airway resistance. In contrast, the change in volume of each subject was associated with the lung volume estimated to cause the closure of the least stable segmental airway of his/her lungs. In addition, the measured drop in FVC post MCh was associated with the estimated volume of gas trapped behind closed segmental airways at RV.

**Conclusions:**

Our data supports the concept that hyperinflation caused by MCh-induced bronchoconstriction is the result of an actively controlled process where parenchymal distending forces on airways are increased to counteract their closure. To our knowledge, this is the first imaging-based study that associates inter-subject differences in whole lung behavior with the interdependence between individual airways and their surrounding parenchyma.

## Introduction

Lung hyperinflation during asthma exacerbations, cause reduced inspiratory capacity and elevation in mechanical load for inspiratory muscles that, in severe cases, lead to muscle fatigue and respiratory [1]. Lung hyperinflation during mechanical ventilation is usually attributed to a passive mechanical origin, namely expiratory flow limitation and elevation of airway resistance and/or breathing rate making the mechanical RC time constant too long in relation to the expiratory time [2]. Consistent with that mechanism would be the observation of increasing lung volume in proportion to the elevation in airway resistance during progressive broncho-provocation in spontaneously breathing subjects [3,4]. Such an increase in lung volume was also found to be concurrent with activation of inspiratory muscles during exhalation [3,5]. Therefore, it is not clear whether hyperinflation in asthma is a passive result of airway obstruction or an active reflex to prevent it. Independent of the mechanisms leading to it, hyperinflation should increase the load to the airway smooth muscle by increasing the tethering forces provided to the airway wall from the surrounding parenchyma [6]. Although the degree to which an increase in lung volume reduces airway narrowing during bronchoconstriction remains unclear [7] the effect was recently demonstrated with in-vitro preparations of human airways surrounded by parenchyma [8] and by a computational model of a branching tree structure imbedded in expanding parenchyma [9]. That model demonstrated emergence of large ventilation defects when airway smooth muscle activation increased above a critical level, explaining the patchiness in regional ventilation observed in asthma [10]. The model also showed that elevation of global lung volume increased the critical smooth muscle activation required for the onset of airway instability [11]. Mead et al. [12] estimated that the expanding parenchymal stress around an airway could be equal or greater than local transpulmonary pressure. This approximation may be applicable to normal airways at baseline conditions and to some degree to homogeneous airway narrowing but not likely the extreme heterogeneity in airway narrowing during bronchoconstriction with ventilation defects surrounded by well-ventilated regions.

Despite experimental observations and theoretical predictions, there has been a lack of *in-vivo* evidence demonstrating a link between airway-parenchyma interdependence, and heterogeneous airway constriction in humans.

Here we present a novel experimental approach to characterize the interaction of individual human airways and their surrounding parenchyma. We used high-resolution computed tomography (HRCT) derived quantification of the degree of narrowing of individual airways and measured their distension during lung inflation relative to that of their surrounding parenchyma. With such a data we preceded to test whether the degree in elevation of lung volume following broncho-provocation amongst subjects were associated with corresponding degree in airway narrowing or, alternatively, were a subject-specific response aimed at preventing the onset of airway closure. The imaging data was also used to evaluate whether the reductions in FVC of the subjects could be accounted by the closure individual segmental airways within their lungs.

## Methods

This research was approved by the *Massachusetts General Hospital Institutional Review Board* (Protocols 2007P000493 and 2017P000326). The clinical investigation was conducted according to the principles expressed in the Declaration of Helsinki and informed consent, was obtained from the participants.

### Theoretical framework

**Fig 1:**
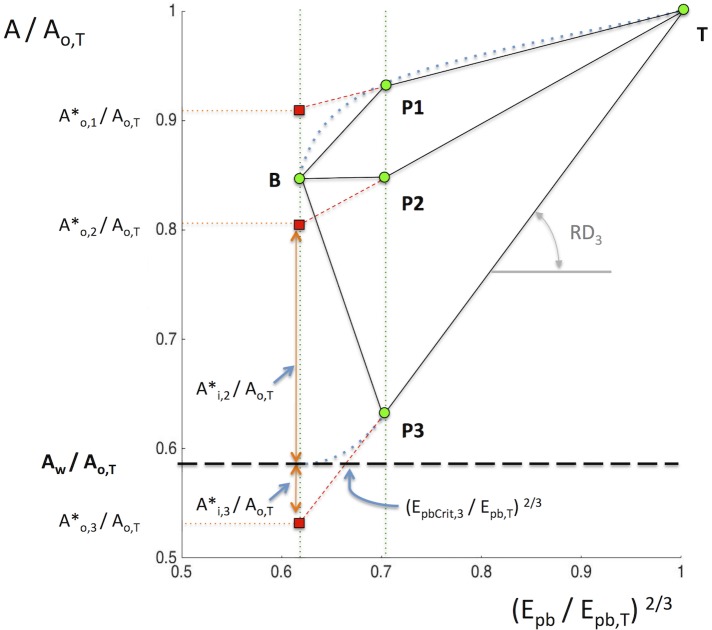
Empirical framework, (for explanation see text).

We characterize the interplay between the airway wall and its surrounding parenchyma in terms of the relationship between outer airway cross-sectional area (*A*_*o*_) and the degree of surrounding (peribronchial) parenchymal expansion (*E*_*pb*_) elevated to the ^2/3^ power *(E*_*pb*_^*2/3*^) to preserve dimensionality. *A*_*o*_ is the sum of inner luminal cross sectional area, A_i_, plus the cross sectional area of the airway wall, *A*_*w*._ *E*_*pb*_ is defined as the volumetric ratio of gas to parenchyma tissue within a spherical region adjacent to the airway [13–17] as detailed in the supporting material (S1 Fig). To account for size differences in among different airways, the relationship is expressed as a plot where *A*_*o*_ and *E*_*pb*_^*2/3*^ are normalized by their respective values measured at total lung capacity (TLC) or: (*A*_*o*_/*A*_*o*,*T*_) vs (*E*_*pb*_/*E*_*pb*,T_)^2*/3*^ (Fig 1). In such a plot lung expansion from mean lung volume (MLV) to TLC for a given airway, follows a path with a cord slope that defines the distensibility of the airway relative to that of the peribronchial parenchyma, *RD*:

$$RD=\frac{1-\frac{A_{o,P}}{A_{o,T}}}{1-\frac{{E_{pb,P}}^{2/3}}{{E_{pb,T}}^{2/3}}}$$ (1)

Airways with *RD* < 1 are relatively stiffer, and thus expands less than the surrounding parenchyma as lung volume is increased to TLC. Conversely, airways with *RD* > 1 expand relatively more than the surrounding parenchyma and those with *RD* = 1 have a stiffness equivalent to that of the parenchyma.

Examples for the behavior of three hypothetical airways are presented in Fig 1. All three airways start with a fully relaxed state at baseline (point B) but are submitted to different degrees of smooth muscle activation during broncho-provocation. It is also assumed that lung volume increases following broncho-provocation such that the peribronchial parenchyma increases normalized expansion (*E*_*pb*_/*E*_*pb*,T_)^2*/3*^ from 0.62 to 0.7. Depending on their degree of smooth muscle activation, the states of the three airways should follow paths to different equilibrium states following bronchial challenge. If the smooth muscle force is not affected by the MCh challenge, the airway state can be expected to follow the non-linear path such as that for a fully relaxed airway [18] reaching an equilibrium state at P1, with *A*_*o*,*P1*_/*A*_*o*,*T*_ > *A*_*o*,*B*_/*A*_*o*,*T*_. With further expansion of the lung to TLC, the airway becomes stiffer than the parenchyma (*RD* < 1), as it approaches its maximum passive circumference. In a second airway, the force generated by the airway smooth muscle (ASM) after MCh is just enough to balance the force exerted by the expanded parenchyma, and *A*_*o*_/*A*_*o*,*T*_ remains unchanged, reaching its new state P2. In a third airway, the ASM force greatly exceeds the forces generated by the expansion of the surrounding parenchyma, and *A*_*o*_/*A*_*o*,*T*_ becomes smaller in state P3 or, *A*_*o*,*P3*_/*A*_*o*,*T*_ < *A*_*o*,*B*_/*A*_*o*,*T*_. Further elevations of lung volume to TLC for these last two airways results in paths of higher *RD* (slope) than that for the first airway.

Assuming linearity for the *A*_*o*,*P*_/*A*_*o*,*T*_ vs. (*E*_*pb*_/*E*_*pb*,*T*_*)*^*2/3*^ *relationship for each airway*, one can estimate the outer cross-sectional area that any airway would have reached (A*_o_*/A*_*o*,*T*_), had the peribronchial lung expansion remained unchanged after MCh. Such an assumption seems reasonable for constricted airways based on CT imaging observations of canine lungs by Brown et al. [18] (S7 Fig).

However, Brown et al. [18] also showed that for a relaxed airway the slope of the relationship decreases with lung inflation and thus for those airways A*_o,*1*_*/A*_*o*,*T*_ would over-estimate the expected value of the airway that should have remained equal to that at baseline *A*_*o*,*B*_*/A*_*o*,*T*_.

Nonetheless, for airways with constricting ASM, such as those in conditions P2 and P3, the extrapolated values of A*_o_ */A*_*o*,*T*_ would be lower than at baseline, *A*_*o*,*B*_*/A*_*o*,*T*,_ and post MCh, *A*_*o*,*P*_*/A*_*o*,*T*_. Note also that for an airway with very high ASM force, in a condition like P3, the extrapolated value A*_o_ may be less than *A*_*w*_, or (A*_o,*3*_*/A*_*o*,*T*_ < *A*_*w*,*P*_*/A*_*o*,*T*_). Since the luminal area *A*_*i* =_ *A*_*o*_ + *A*_*w*_, this would predict a negative value for *A*_*i*_ or:

$$A_{i,3}^{*}/A_{o,T}=(A_{o,3}^{*}/A_{o,T}-A_{w,P}/A_{o,T})<0$$

Clearly, a negative luminal area would be physically impossible. However a negative value of A*_i,*3*_*/A*_*o*,*T*_ would represent a state where ASM force more than exceeds the distending force opposed by the parenchyma distended at baseline conditions, and thus the airway lumen would be completely shut, and the excess in ASM force would be balanced by compression forces on airway wall tissues central to the ASM band. In this case, as the airway would remain closed as lung volume increases until peribronchial expansion exceeds a critical value (*E*_*pbCrit*,*3*_/*E*_*pb*,*T*_*)*^*2/3*^.

Evaluation of the critical peribronchial expansion for each segmental airway of the tree and assuming that the airway wall areas remain unchanged during an expiratory forced vital capacity (FVC), allows estimation of the volume of the subtended lung that would be trapped by closing airways during the expiratory maneuver. The sum of these estimated trapped volumes during a FVC maneuver would be an approximation of the contribution of these closing segmental airways to the reduction in FVC after bronchoconstriction.

In summary, in this report we used the empirical framework described above for the following objectives: 1) Analyze experimental imaging data from normal and asthmatic lungs in conditions where the subjects are allowed to choose their mean breathing volume before and after bronchoconstriction. 2) Evaluate whether closure of individual segmental airways could have occurred if lung volume was not elevated following MCh bronchoconstriction (were there airways with values of A*_i,*3*_*/A*_*o*,*T*_ <0 ?). 3) Test whether the elevation of lung volume by each subject following bronchoconstriction resulted in peribronchial expansion around segmental airways that matched or exceeds the critical value for closure. And 4) estimate the volume of gas expected to be trapped behind closed segmental airways during a forced vital capacity maneuver and compared it with the changes in FVC observed for each subject. Specific imaging protocols, analysis of the imaging data, and methods for comparison of predicted against measured values for each subject are presented below.

### Subjects

This study analyzed HRCT scans of subjects that were imaged with PET/CT as part of published research protocols [19,20]. Data was collected under a Massachusetts General Hospital IRB approved protocol. Scans from seven subjects with demonstrated reversible obstruction and mild-moderate asthma (AS) and nine healthy controls (NA) were analyzed. AS subjects were selected according to the criteria of the NIH Global Initiative for Asthma [21]: airways hyperresponsiveness, a history of intermittent airway obstruction, less than daily symptoms, forced exhaled volume within 1 sec (FEV_1_) or forced vital capacity (FVC) ≥80% predicted, and peak flow or FEV1 variability of ≤30%.

At the time of the study all subjects were either non-smokers or had a pack-year smoking history ≤ 10. Other exclusion criteria were: known cardiopulmonary disease or chronic respiratory disorder other than asthma, pregnant or nursing women (confirmed via serum beta-HCG test), upper and lower airway respiratory infection or emergency visits or hospitalizations in the month prior to screening, previous exposure to more than half the radiation dose for the study in the past year (3.75 mSv, or equivalent), or had taken oral steroids in the past year for their asthma. No systemic or inhaled corticosteroids had been used within one week prior to enrollment.

### Study design

Subjects attended two separate visits. Short- and long-acting bronchodilator medications were stopped 8 and 48 hours respectively, prior to the visits. In a screening visit, baseline spirometry was acquired in the erect position (Satellite Spirometer, Jones Medical Instrument, USA) followed by a standard five-breaths MCh challenge test with a jet nebulizer (Rosenthal dosimeter) according to ATS guidelines [22]. AS subjects were given MCh at increasing concentrations to estimate the value that caused a 20% reduction in FEV_1_ (PC20). For AS subjects PC20 dose ranged form 0.116 to 4.9 mg/ml. In NA subjects the absence of airway hyperresponsiveness was ascertained when a MCh challenge with a maximum concentration of 25 mg/ml failed to reduce FEV1 by 20% from baseline. During a second (imaging) visit, the subject was positioned supine in the PET/CT scanner and a baseline (*B*) full-chest HRCT scans was obtained during a short breath-hold (~12 sec) at a lung volume equal to the mean lung volume (MLV) measured during a 30 sec of stable tidal breathing prior to the scan.

In order to determine the values of MLV, and ensure constant volume during the breath-holds, impedance plethysmography (SomnoStarPT, SensorMedics, USA) was used to monitor dynamic lung volume in real time. The SomnoStarPT output signal was plotted in real time with a laptop computer and the screen presented to the subject via a head mount display (Argo Cinema 2 Goggles, Welton Electronics Inc, Hong Kong). MLV was estimated by averaging the volume signal over 30 seconds of stable breathing, and presented to the subject as a reference line superimposed on the real time lung volume signal.

Following the *baseline* scan (*B*), the MCh challenge was given at a concentration equal to the previously determined PC20 for the AS subjects, and equal to 25 mg/ml for the NA subjects.

**Fig 2:**
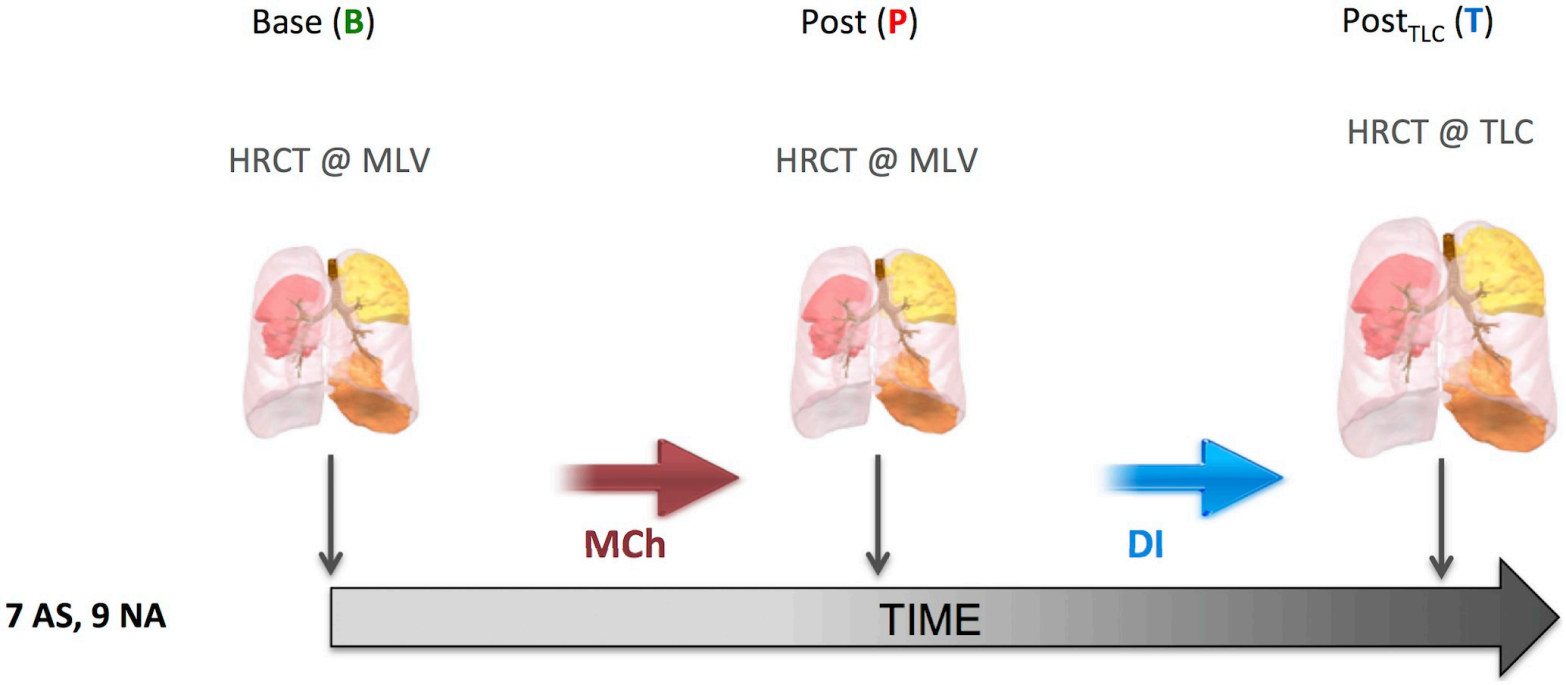
Study protocol. With the subjects in the supine position, HRCT images were acquired during a short breath hold (~12 s) under three consecutive conditions: *1*) at baseline (*B*) and imaged at mean lung volume (MLV), where MLV was defined as the average lung volume during stable tidal breathing; 2) following five deep breaths of MCh at his/her previously determined PC20 dose and imaged at the subject’s spontaneous MLV (*P*); and *3*) post MCh and imaged at total lung capacity (*T*). Colored regions of the lung correspond to 3 of the ~19 parenchyma segmental regions studied in each lung.

After allowing 10 minutes to establish bronchoconstriction, a second post MCh HRCT scan (*P*) was obtained at a lung volume equal to the subject’s new spontaneous MLV. Finally, and still during bronchoconstriction, a third HRCT scan (*T*) was acquired with the lungs at total lung capacity (Fig 2). Spirometry was performed after *B* and *P* scans with the subject remaining supine within the scanner.

In order to standardize the lung volume history and facilitate the breath-hold during imaging, prior to the B and P scans, the subjects were asked to take a deep inspiration and exhale out stopping at the MLV value shown at the head mount display.

Analysis showed no difference in lung function at baseline between the two groups although the AS group was, by definition, hyper-responsive, as indicated by a PC20 < 4 mg/ml [22]. FEV_1_ and FVC values were influenced by the change in posture between the screening visit (seated) and the imaging day (supine) with FEV_1_ significantly higher in the screening visit both the asthma (p<0.05) and control groups (p<0.001) (Table 1).

**Table 1:** Subject characteristics.

Group (M:F)	Control (4:5)	Asthma (5:2)	Unpaired T-Test
Age, years	31.7±10.3	31.4±11.7	
Height, cm	165.7±6.4	173.3±7.1	P<0.05
Weight, kg	64.9±7.8	76.3±13.6	P = 0.05
*Screening Visit (seated)*			
*Baseline*			
FEV1, L (% predicted)	3.3±0.7 (93%)	3.5±0.5 (88%)	
FVC, L (% predicted)	4.0±0.9 (93%)	4.6±0.8 (93%)	
PC20, mg/mL	>25	2.3±1.9	P<0.001
*Imaging Visit (supine)*			
*Baseline (B)*			
FEV_1_, L (% predicted)	2.8±0.6 (80%)***	2.9±0.3 (74%)*	
FVC, L (% predicted)	3.4±0.8 (80%)***	4.0±0.5 (82%)*	
MLV, L	2.7±0.4	3.3±0.8	
*Post MCh (P)*			
FEV_1_, L (% predicted)	2.1 ± 0.6 (60%)^†††^	2.0 ± 0.5 (50%)^††^	
FVC, L (% predicted)	3.0 ± 0.9 (69%)^††^	2.9 ± 0.5 (60%)^††^	
MLV, L	3.4±0.3^††^	4.1±1.0^†^	
TLC, L	5.2±0.7	5.6±1.1	

Paired t-test comparison between baseline seated and baseline supine;

* p<0.05,

*** p<0.001.

Paired t-test comparison between baseline and post methacholine;

^†^ p<0.05,

^††^ p<0.01,

^†††^ p<0.001

### Imaging and regional analysis

The HRCT scans were obtained using a Siemens Biograph 64 PET-CT scanner. The scanner was used in a helical mode with 64 slices per rotation, 0.6 mm collimation, a pitch of 1, 120 kV peak, and 80 mA. The image reconstruction was done using the B31 kernel with a slice thickness of 0.75 mm, 0.5 mm slice increment and 0.25 mm overlap (as recommended by VIDA Diagnostics, IA, USA).

#### Airway and parenchymal segmentation and measurements

**Fig 3:**
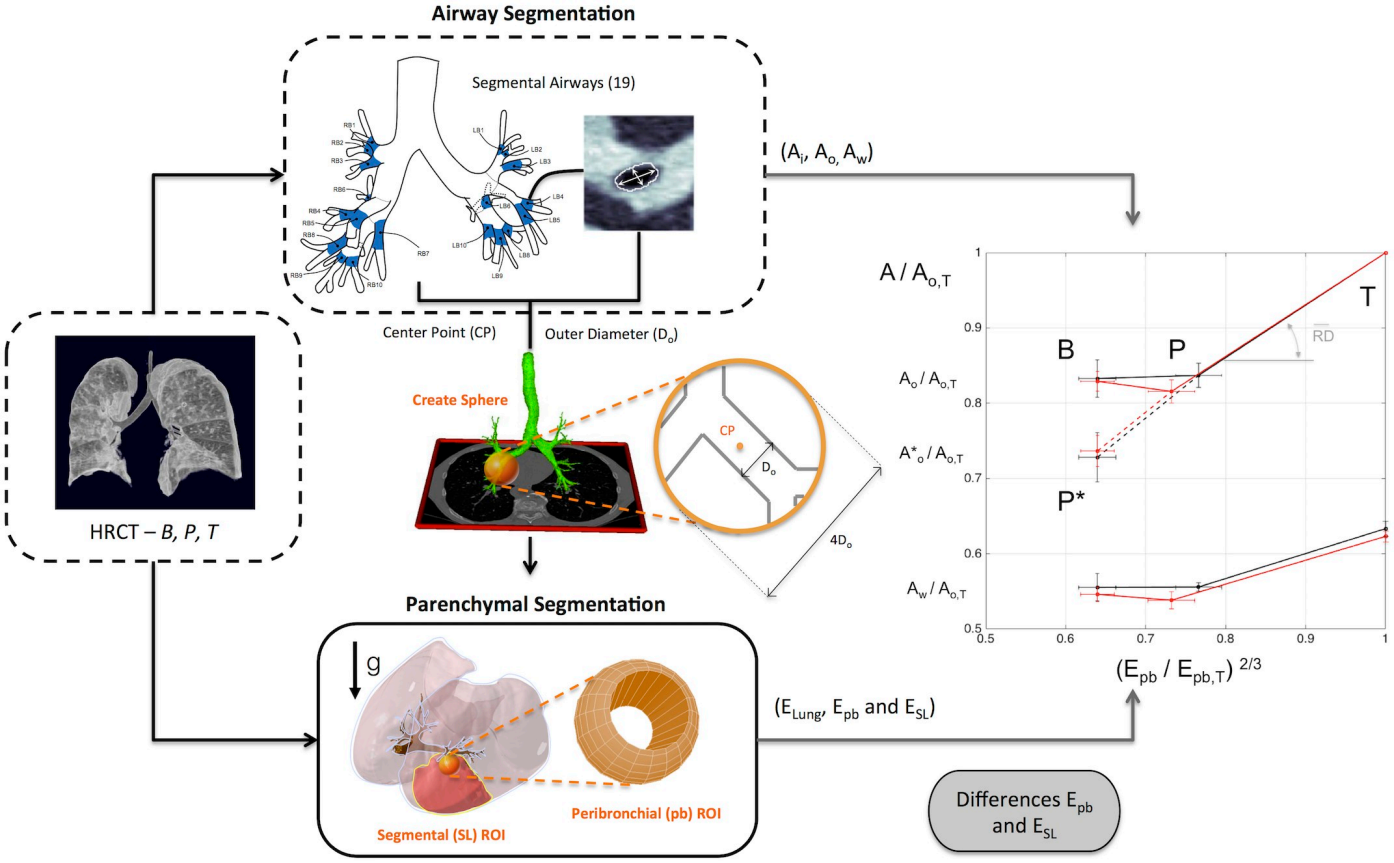
Methodology overview. Airway and parenchymal segmentation were performed to the HRCT images at the three different conditions. Using VIDA software, values of inner (*A*_*i*_), outer (*A*_*o*_) and wall (*A*_*w*_) cross-sectional areas were obtained for each of the 19 segmental airways. A spherical region of diameter four times the outer airway diameter was defined around each airway center point. Intersection of the sphere with the parenchymal mask defined a peribronchial (pb) ROI, and its average expansion (*E*_*pb*_) normalized by its value at TLC (*E*_*pb*_ /*E*_*pb*,*T*_), was plotted vs *A*_*o*_ normalized by the outer airway area at TLC (*A*_*o*_ /*A*_*o*,*T*_). The data shown in the plot are averages for all AS (black) and NA (red) subjects. (One graphical element on this Fig is a modified version from original in http://image.diku.dk/exact/).

Image analysis included quantification of airway dimensions and regional parenchymal expansion (Fig 3). Airways and parenchymal regions were rendered, segmented and characterized from the HRCT scans using the Pulmonary Workstation 2.0 (PW2) software (VIDA Diagnostics, IA, USA). Airway dimensions were extracted from PW2 for 19 anatomically defined segmental airways for each of the three conditions [23]. The following parameters were imported into MALTAB (Mathworks, Natick, MA) for each airway: 1) lumen (*A*_*i*_) and 2) outer (*A*_*o*_) cross-sectional areas, 3) length (*L*), 4) centerline, 5) branching point information (between mother and daughter airways for each bifurcation), and 5) anatomical labeling. Both *A*_*i*_ and *A*_*o*_ corresponded to the average across sections of the middle third of the airway length, as defined by PW2 [23]. By inspection, airways with centerline not perpendicular to the airway cross-section and/or showing software segmentation errors were excluded (typically one airway per scan). Only airways that could be identified and characterized in each of the three scans were included in the analysis.

Using PW2 on the HRCT images, we also defined masks of the lung pleural surfaces, airway tree, vessel tree and the 19 segmental sub-lobar regions (subtended by the 19 identified segmental airways, Fig 3). The rendered airways and segmental sub-lobar regions were visual inspected for completeness and accuracy. Manual editing of improper segmentation was performed when necessary.

A **lung parenchyma mask** was obtained after subtracting from the lung surface masks voxels including airway and pulmonary vessel trees, and applying a threshold including voxels between -950 to -400 Hounsfield units (HU). This excluded bronchial, pulmonary vessel, and lymphatic tissues, (especially those located within the interlobular septa), and nerves, that were obvious by close inspection. For each voxel the fractional air content or gas fraction (*F*_*gas*_) is calculated as in reference [24] using the equation

$$F_{gas}=({HU}_{blood}-HU)/({HU}_{blood}-{HU}_{air})$$

With *HU*_*blood*_ = 65 and *HU*_*air*_ = -1,000, and *HU* in Hounsfield Units.

**Segmental parenchyma** ROI’s were defined as the intersection of the segmental regions with the lung parenchymal mask.

**Peribronchial parenchyma ROI’s** were defined for segmental airways as follows. First, the location of the center point (CP) along the axis of each segmental airway was defined. For the *B* condition, a spherical region, centered at the CP, was defined with a diameter equal to four times that of an equivalent circle with area *A*_*o*_. The intersection of this sphere with the parenchyma mask defined a peribronchial ROI. The relative diameter of the sphere was defined based on modeling studies showing non-uniform parenchymal deformation within this region during lung inflation [25]. For the *P* and *T* conditions, the diameter of the sphere was adjusted iteratively, until the total parenchymal tissue volume within the ROI was within 5% of that measured at *B*. This ensured that approximately the same tissue was evaluated for the three conditions.

For each airway, the following parameters were also determined: 1) The relative vertical-distance (Δ*h*) between the airway’s CP and the corresponding geometric center of the subtended segmental parenchymal ROI normalized by the lung maximal dorso-ventral height. 2) The **parenchyma expansion** of the subtended segment (*E*_*SL*_) and the **peribronchial parenchyma expansion** (*E*_*pb*_*)*, both estimated as the ROI’s voxel average of: *F*_*gas*_ /(1-*F*_*gas*_), which correspond to the average voxel **gas-to-tissue volume ratio**. 3) The sampled volume by the peri-bronchial parenchyma ROI (*Fs*), as a fraction of the theoretical ROI volume (the volume of the sphere minus that of an intersecting cylinder of cross-sectional area *A*_*o*_).

For each condition, the global lung expansion (*E*_*Lung*_) was estimated as the voxel average of gas-to-tissue volume ratios within the global lung parenchymal mask.

#### Vertical dependence of regional differences in parenchymal expansion

For segmental airways the difference between peribronchial and subtended segmental parenchymal expansion (*ΔE = E*_*SL*_ − *E*_*pb*_) was evaluated. *ΔE* showed a systematic dependency to their vertical relative distance, *Δh*. *Such a dependency* was characterized by linear regression for airways of each subject and condition.

Also for each subject and condition, the average difference between segmental and peribronchial expansion, (*ΔE*_*mean*_) was calculated after adjusting for the vertical distance dependency of each subject/condition, and the relationship between *ΔE*_*mean*_ and *E*_*Lung*_ evaluated by linear regression for the 3 conditions (represented in figures of results section ‘changes in luminal cross-sectional area, mean breathing lung volume and forced vital capacity with methacholine’).

#### Iso-volume airway outer area and critical peribronchial expansion

Assuming a linear relation between normalized outer airway area (*A*_*o*_) peribronchial expansion (*E*_*pb*_), and hence a constant RD for each segmental airway, the iso-volume airway cross-sectional outer area ratio (A*_o_*/A*_*o*,*T*_) that would have been reached, had the peribronchial lung expansion remained unchanged with MCh (Fig 1), was calculated as:

$$A_{o}^{*}/A_{o,T}=\left(\frac{A_{o,P}}{A_{o,T}}-RD(\frac{{E_{pb,P}}^{2/3}-{E_{pb,B}}^{2/3}}{{E_{pb,T}}^{2/3}})\right)$$ (2)

The critical peribronchial expansion for airway closure (when *Ao = Aw*) of each airway (*E*_*pbCrit*_*/E*_*pb*,*T*_) was calculated assuming that *A*_*w*_ was equal to that measured at MLV post MCh (*A*_*w*,*P*_):

$$\left(\frac{E_{pbCrit}}{E_{pb,T}}\right)={\left({\left(\frac{E_{pb,B}}{E_{pb,T}}\right)}^{\frac{2}{3}}-\frac{\left(\frac{A_{o}^{*}-A_{w,P}}{A_{o,T}}\right)}{RD}\right)}^{\frac{3}{2}}$$ (3a)

Given that *A*_*w*_ was found to be greater at TLC compared with MLV, during a FVC the critical expansion the gas volume trapped behind closing segmental airways, was estimated assuming that *A*_*w*_ equal to that measured at TLC (*A*_*w*,*T*_):

$${{\left(\frac{E_{pbCrit}}{E_{pb,T}}\right)}_{FVC}=(1-\frac{\left(1-\frac{A_{w,T}}{A_{o,T}}\right)}{RD})}^{\frac{3}{2}}$$ (3b)

### Change in mean lung volume and forced vital capacity after MCh

**Fig 4:**
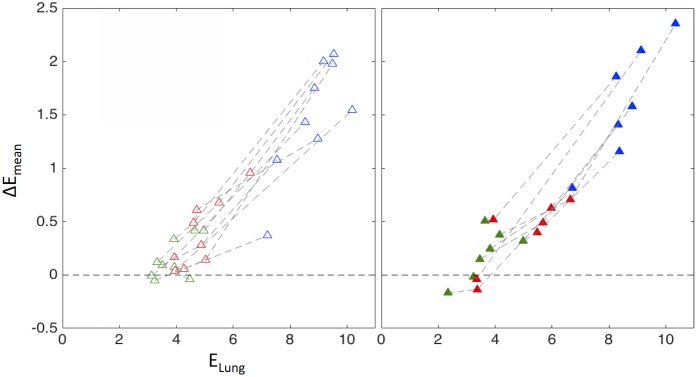
Mean difference between sub-lobar and peribronchial parenchyma expansion, adjusted by height difference, *ΔE*_*mean*_ plotted for each subject vs. average lung expansion, *E*_*Lung*_, in three conditions for the group of subjects without asthma (NA, left) and with asthma (AS, right). The conditions are Baseline at MLV (Green), post MCh at MLV (Red), and post at TLC (Blue). Points for each subject are connected with dashed lines. There is no apparent difference in the behavior of both groups.

To evaluate the gas volume trapped behind each closing airway using Eqs 3a or 3b, it was necessary to account for a measured difference *ΔE*_*mean*_ between segment’s distal parenchyma, *E*_*SL*_, and its local peribronchial expansions *E*_*pb*_, using the empirical relationship obtained between *ΔE*_*mean*_ and *E*_*Lung*_ presented in Fig 4, as:

$$\frac{\overset{-}{E_{SL}}}{\overset{-}{E_{pb}}}=\frac{1-\frac{{\Delta E}_{mean,P}}{E_{Lung,P}}}{1-\frac{{\Delta E}_{mean,T}}{E_{Lung,T}}}$$ (4)

For each subject we calculated an expected change in lung volume between baseline and that at which the first segmental airway would have closed (*ΔV*_*C*_). To do that, we selected the airway with the lowest value of (*A*_i_*/A_*o*,*T*_) and estimated its critical peribronchial expansion *E*_*pbCrit*_*/E*_*pb*,*T*_, using the empirical relationship between peribronchial and segmental expansion for that subject (Eq 4), *ΔV*_*C*_ was estimated as:

$$\frac{\Delta V_{c}}{TLC}=(\frac{E_{pbCrit}}{E_{pb,T}}-\frac{E_{pb,B}}{E_{pb,T}})*\frac{\overset{-}{E_{SL}}}{\overset{-}{E_{pb}}}$$ (5)

TLC normalized values of *ΔV*_*C*_ were plotted against the change those MLV measured for each subject (see Results).

Finally, to estimate the total sum of air volume trapped behind closing airways (*V*_*Tr*_) during a FVC maneuver, we considered the sum of volumes behind each airway (*j*) expected to close during a forced vital capacity, or those behind airways with *E*_*pbCrit*_ > *E*_*pb*_ at RV ($E_{pb,RV}=\frac{{RV}_{P}}{TLC}/\frac{\overset{-}{E_{SL}}}{\overset{-}{E_{pb}}}$):

$$\frac{V_{Tr}}{{FVC}_{B}}=\frac{\sum_{j}({\left(\frac{E_{pbCrit}}{E_{pb,T}}\right)}_{j}/\frac{\overset{-}{E_{SL}}}{\overset{-}{E_{pb}}})-\frac{{RV}_{P}}{TLC}}{{FVC}_{B}}$$ (6)

were the residual volume at RV post MCh, RV_P_, was calculated as the difference between TLC (measured from the CT) and FVC, assed by spirometry post MCh in the supine position.

$\frac{V_{Tr}}{{FVC}_{B}}$ was then plotted against the fractional reduction of FVC measured between baseline and post MCh.

### Statistical analysis

Data are expressed as mean ± SD and statistical analysis was completed using MATLAB. Significance was accepted at P < 0.05. Paired T-tests were used for assessing differences between conditions, or between peribronchial and segmental measures for each subject, and unpaired T-tests were used to assess differences in demographic and measurements between the asthma and control groups.

## Results

### Differences between peribronchial and segmental expansion

Histograms of voxel gas-to-tissue volume fraction (*E*) values within peribronchial and segmental ROI’s showed a shift to the right as lung volume increased from baseline to post MLV and to TLC (S2 Fig).

In average MLV as a fraction of TLC increased from 0.53±0.78, at baseline, to 0.66±0.11 after bronchoconstriction (p<0.01) in NA and from 0.55±0.97 to 0.68±0.12 in AS (p<0.05), but these changes were not significantly different between groups (Tables 2 and 3). Consistent with the changes in lung volume, the average values of *E*_*pb*_ and *E*_*SL*_, also increased after MCh challenge, and further increased with lung inflation to TLC. The changes in *E*_*SL*_ for the subtended segment’s peripheral parenchyma were systematically greater than those in peribronchial *E*_*pb*_ of the feeding airway (S3 Fig).

**Table 2:** Differences between peribronchial and segmental parenchymal expansion *ΔE*.

Group	AS			NA		
Condition	B	P	T	B	P	T
V_L_, %TLC	54.8±9.6	68.3±12.1*	100^†††^^,^^‡‡‡^	53.1±7.8	65.9±10.9**	100^†††^^,^^‡‡‡^
***ΔE vs*. *Δh***						
R^2^	0.59±0.21	0.47±0.18	0.22±0.12^†^^,^^‡‡^	0.68±0.07	0.60±0.10* ^ɸ^	0.39±0.20^†^^,^^‡‡^^,^ ^ɸ^
Slope	3.02±1.45	3.12±1.43	2.69±0.83	3.18±0.52	2.93±0.49	3.68±1.11^ɸ^
Intercept	0.20±0.23	0.37±0.33*	1.61±0.54^†††^^,^^‡‡‡^	0.15±0.19	0.38±0.32**	1.50±0.55^†††^^,^^‡‡‡^
*Δh*	0.017±0.015	0.017±0.017	0.013±0.014	0.026±0.011	0.029±0.011	0.025±0.014
***ΔE***_***mean***_ ***vs*. *E***_***Lung***_						
*ΔE*_*mean*_	0.20±0.23	0.37±0.33*	1.61±0.54^†††^^,^^‡‡‡^	0.15±0.19	0.38±0.32**	1.50±0.55^†††^^,^^‡‡‡^
*E*_*Lung*_	3.66±0.81	4.92±1.34**	8.56±1.09^†††^^,^^‡‡‡^	4.10±0.62	4.86±0.85*	8.77±1.06^†††^^,^^‡‡‡^
R^2^	0.54	0.72	0.79	0.44	0.70	0.64
Slope	0.21	0.21	0.44	0.19	0.32	0.45
Intercept	-0.57	-0.65	-2.13	-0.59	-1.17	-2.50

B, baseline; P, post MCh challenge; T, total lung capacity; AS, asthmatics; NA, non-asthmatics; *ΔE*, difference in parenchyma expansion between segmental and peribronchial; *Δh*, relative vertical distance; *E*_*Lung*_, average lung parenchyma expansion; *ΔE*_*mean*_, average difference between segmental and peribronchial expansion after accounting for the vertical location dependence; m, slope. See Abbreviations.

Paired t-test comparison between baseline and post;

* p<0.05,

** p<0.01,

*** p<0.001.

Paired t-test comparison between post and total lung capacity at post;

^†^ p<0.05,

^††^ p<0.01,

^†††^ p<0.001

Paired t-test comparison between baseline and total lung capacity at post;

^‡^ p<0.05,

^‡‡^ p<0.01,

^‡‡‡^ p<0.001

Unpaired t-test comparison between AS and NA;

^ɸ^ p<0.05,

^ɸɸ^ p<0.01,

^ɸɸɸ^ p<0.001

**Table 3:** Differences between peribronchial and segmental parenchymal expansion *ΔE (Continued)*.

Group	AS		NA	
Condition	B-P	P-T	B-P	P-T
***ΔE*_*mean*_*vs*. *E*_*Lung*_**				
Change in *ΔE*_*mean*_	0.16±0.18	1.25±0.51^¥¥^	0.23±1.19	1.12±0.50^¥¥¥^
Change in *E*_*Lung*_	1.26±1.01	3.65±1.13^¥¥^	0.93±0.88	4.00±1.22^¥¥¥^
m	0.07±0.14	0.34±0.06^¥¥^	0.47±1.04	0.28±0.10

B, baseline; P, post MCh challenge; T, total lung capacity; AS, asthmatics; NA, non-asthmatics; *ΔE*, difference in parenchyma expansion between segmental and peribronchial; *E*_*Lung*_, average lung parenchyma expansion; *ΔE*_*mean*_, average difference between segmental and peribronchial expansion after accounting for the vertical location dependence; m, slope. See Abbreviations.

Paired t-test comparison between bronchoconstriction by MCh (B-P) and deep inhalation (P-T);

^¥^ p<0.05,

^¥¥^ p<0.01,

^¥¥¥^ p<0.001

This difference in parenchymal expansion between average subtended segment parenchyma and peribronchial parenchyma ROIs, was heterogeneous between segments but presented a systematic dependency with the vertical-distance between the respective ROI’s geometric centers for all conditions (S4 Fig): *ΔE = E*_*SL*_*-E*_*pb*_ was greater for segmental airways feeding non-dependent parenchyma (*Δh>0*) and lower for airways feeding more dependent parenchyma (*Δh<0*).

The average difference between segmental and peribronchial expansion, after de-trending for the vertical location dependence *ΔE*_*mean*_, was highly dependent on the average expansion of the whole lung (*E*_*Lung*_), for each subject and condition and for both AS and NA subjects. No significant differences between AS and NA were found in this dependence but *ΔE*_*mean*_ tended to be higher for AS at TLC.

Note that for both groups, *ΔE*_*mean*_ was around zero in the subjects-conditions with the lowest values of *E*_*Lung*_, and that *ΔE*_*mean*_ increased monotonically as *E*_*Lung*_ increased (represented in figures of section ‘changes in luminal cross-sectional area, mean breathing lung volume and forced vital capacity with methacholine’).

### Behavior of airways and peribronchial parenchyma during bronchoconstriction

**Fig 5:**
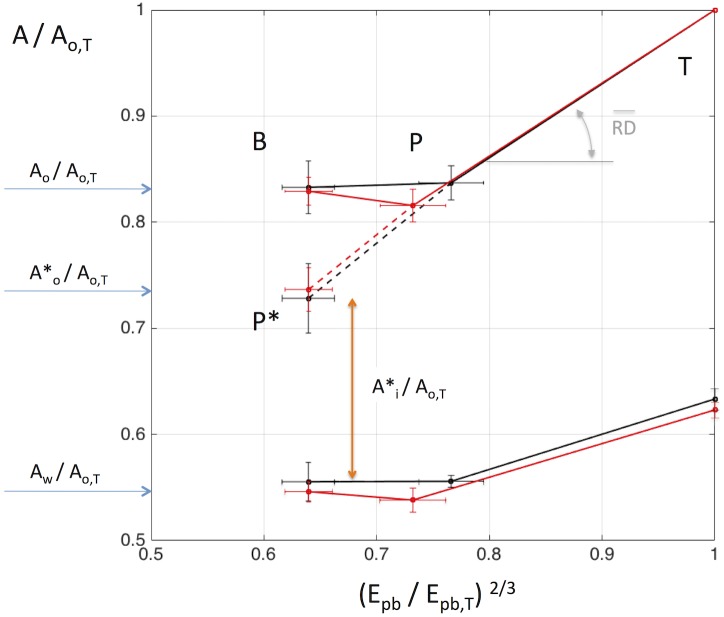
Effects of broncho-provocation with MCh (B to P) and inhalation to TLC (P to T), on average airway cross-sectional outer area (*A*_*o*_), vs the corresponding averages of peribronchial parenchymal expansions, *E*_*pb*_. Plots present the relationship between, A_O_ normalized by the equivalent area at TLC (*A*_*o*,*T*_) and *E*_*pb*_ normalized by *E*_*pb*_ at TLC (*E*_*pb*,*T*_) elevated to the ^2/3^ power, at baseline (*B*), post MCh (*P*) and TLC (*T*) of all segmental airways for all subjects with asthma (AS in in black) and those without asthma (NA in red). The data are presented as mean +/- SEM. The slope of the relationship between P and T corresponds to the relative distensibility (*RD*) of the average airway. $A_{o}^{*}$ corresponds to the value of the airway outer area, *A*_*o*_, as estimated by extrapolation assuming constant RD, that would have been expected had there been no change peribronchial expansion, *E*_*pb*_, following bronchoconstriction. Also shown are the values of airway wall area *A*_*w*_ normalized by *A*_*o*,*T*_ (*A*_*w*_*/A*_*o*,*T*_) for the three states B,P and T, and the difference between A_o_*/A_o,T_ and *A*_*w*_*/A*_*o*,*T*_ corresponds to the average luminal area had peribronchial lung expansion remained the same as at baseline (A_i_*/A_o,T_).

There was no difference between the AS and NA groups for average values of *E*_*pb*_*/E*_*pb*,*T*_ and *A*_*o*_*/A*_*o*,*T*_ at baseline (Fig 5). There was a general increase in mean lung volume (V_L_) following MCh (Tables 2 and 3), as well as in average *E*_*pb*_*/E*_*pb*,*T*_ that increased significantly in both AS and NA groups of subjects (p<0.05 and p<0.01, respectively). Broncho-provocation with MCh left average *A*_*o*_*/A*_*o*,*T*_ unchanged in AS, but caused a minor reduction in NA. Also, the average values of *A*_*o*_*/A*_*o*,*T*_ and *E*_*pb*_*/E*_*pb*,*T*_ for the P*, P and T states fell along the same line for both AS and NA groups, as expected for the similarity in average *RD* between the two groups. Average *RD* 0.75+/-0.48 for AS and 0.75+/-0.55 for NA.

*RD* of individual airways was highly heterogeneous among airways and subjects (S6 Fig). *RD* varied from close to zero in some airways to as high as three in others including values above and below unity.

Average values **of airway wall area** *A*_*w*_ normalized by *A*_*o*,*T*_ (*A*_*w*_*/A*_*o*,*T*_) were similar between B and P but were significantly greater at T in both groups (P<0.001).

### Changes in luminal cross-sectional area (Ai), mean breathing lung volume (MLV) and forced vital capacity (FVC) with methacholine (MCh)

**Fig 6:**
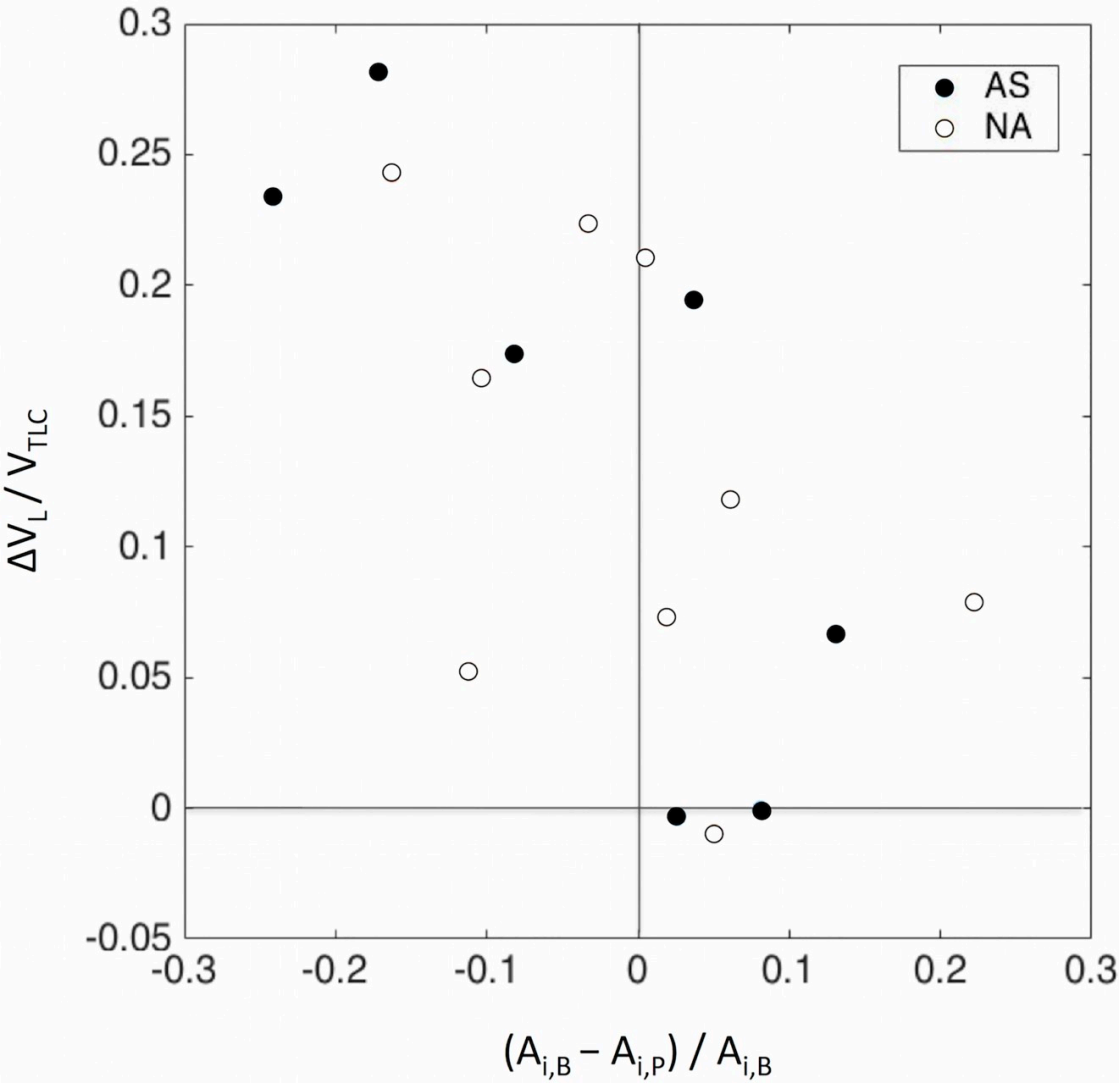
Measured values of *ΔV*_*L*_*/V*_*TLC*_ for each subject *vs*. average change in luminal airway among segmental airways following MCh, for subjects with (AS, blue) and without (NA, red) asthma. Although it was relatively weak, the negative association (R = -0.63, P<0.01 for all subjects) between the variables indicates that subjects with the greatest relative reduction in average airway luminal cross-sectional area, (highest degree of narrowing), tended to be those with the lowest changes in lung volume Post MCh, or inversely: the subjects with greater increases in lung volume Post MCh tended to have an increase in average luminal airway or, (*A*_*i*,*B*_*-A*_*i*,*P*_*)/A*_*i*,*B*_ <0.

There were no statistical differences between AS and NA in the relative degree of obstruction caused by MCh, estimated as the fractional change in airway luminal area between *B* and *P* (*A*_*i*,*B*_*-A*_*i*,*P*_*)/A*_*i*,*B*_. However, the average (*A*_*i*,*B*_*-A*_*i*,*P*_*)/A*_*i*,*B*_ for each subject, was inversely correlated with *ΔV*_*L*_*/TLC*, (R = -0.63, P<0.01 for all subjects, Fig 6). Thus the increase in lung volume between B and P was not greater for the subjects showing the greatest degree of airway narrowing after MCh. On the contrary, the relative reduction in airway lumen caused by MCh occurred in subjects showing the lowest increases in lung volume.

**Fig 7:**
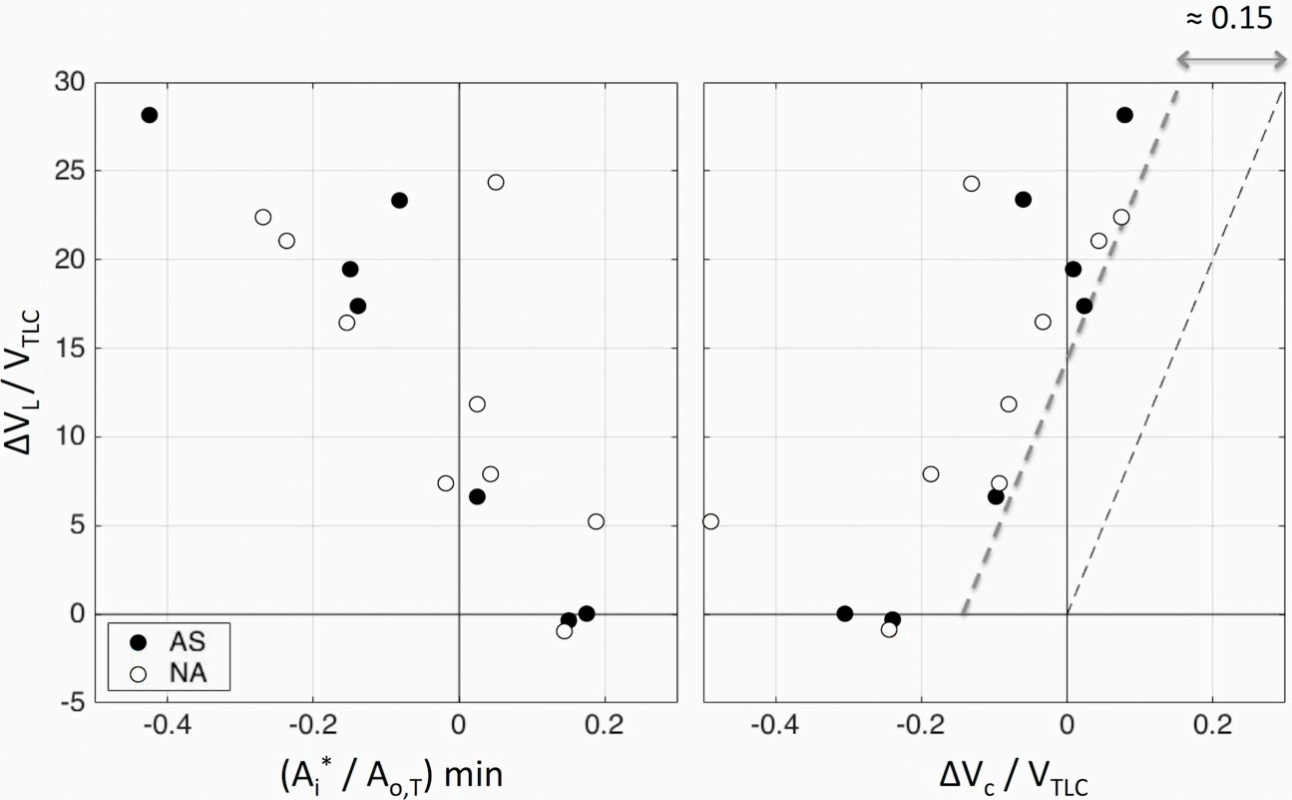
**LEFT:** Plot of the relative change in mean lung volume (*ΔV*_*L*_*/V*_*TLC*_) chosen for breathing after MCh by each subject, *vs* the corresponding minimum value of *A*_i_*/A_*o*,*T*_, amongst all segmental airways of each subject *(A*_i_*/A_*o*,*T*_*)min*. This illustrate an inverse association between the variables (R = -0.83, P<0.001 for all subjects). Each data point corresponds to a subject of the AS (blue) and NA, (red) groups. **RIGHT:** Plot showing the Inverse association (R = 0.76, P<0.001 for all subjects) between *ΔV*_*C*_*/V*_*TLC*_ and the critical change in lung volume causing for first airway to close (*ΔV*_*Crit*_*/V*_*TLC*_) in both groups. Note that the relative change in MLV for each subject after MCh was always at least 15% higher of that for predicting the onset of the first airway closure in each subject.

*ΔV*_*L*_*/ V*_*TLC*_ after MCh, was also inversely correlated (R = -0.83, P < 0.001 for all subjects) with the lowest value of A*/A_*o*,*T*_ of each subject among his segmental airways (A*/A_*o*,*T*_*)min* (Fig 7, left).

Similarly, *ΔV*_*L*_*/V*_*TLC*_, was significantly associated (R = 0.76, P<0.001 for all subjects) with the critical change in lung volume for closure of the first airway normalized by *V*_*TLC*_ (*ΔV*_*C*_*/ V*_*TLC*_) (Fig 7, right). Note that the change in mean lung volume chosen by each subject for breathing after MCh, was at least 15% higher than the critical change in volume estimated for closure of the first airway of that subject.

**Fig 8:**
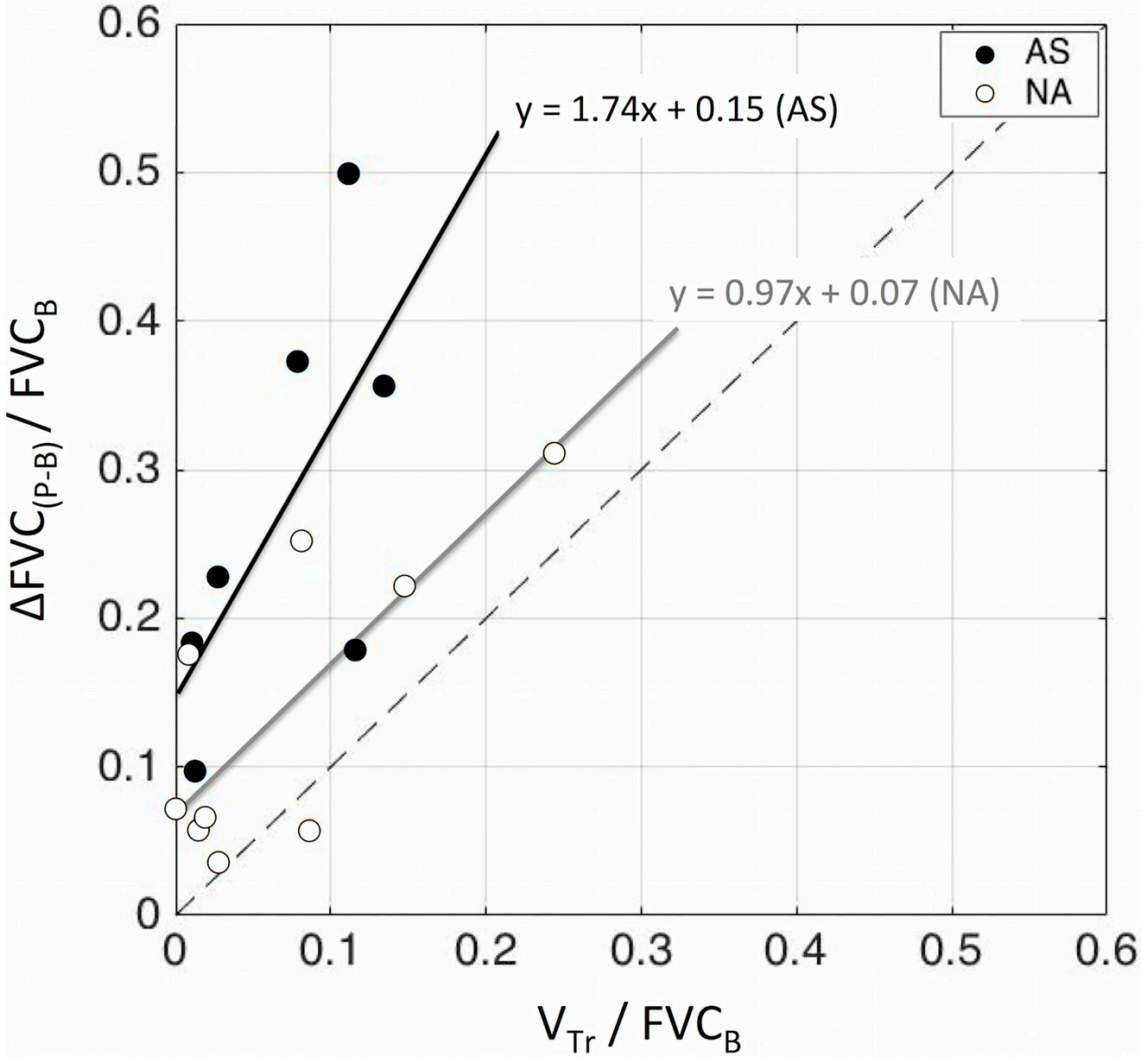
Measured relative drop in FVC from baseline to post MCh (*ΔFVC*_*(P-B)*_*/FVC*_*B*_) plotted as a function of the sum of volumes estimated to be trapped behind closing segmental airways during a force vital capacity maneuver (*V*_*Tr*_ */FVC*_*B*_). Each data point corresponds to a subject of the AS (blue) and NA, (red) groups.

Finally, the change in force vital capacity relative to TLC by MCh (*ΔFVC*_*(P-B)*_*/FVC*_*B*_) for each subject was higher than but correlated with the volume of gas estimated to be trapped behind closed segmental airways (*V*_*Tr*_ */FVC*_*B*_) during a FVC (Fig 8). There were large differences in *ΔFVC*_*(P-B)*_*/FVC*_*B*_ among subjects raging from 3% to 32% of baseline in NA, and from 10% to 50% in the AS group. By enlarge, the measured changes in FVC were equal, or greater, than those predicted by our analysis for all subjects *ΔFVC*_*(P-B)*_*/FVC*_*B*_ ≥ *ΔV*_*Tr*_*/ FVC*_*B*_.

In the NA subjects *ΔFVC*_*(P-B)*_ increased in the same proportion of the estimated trapped gas volumes (slope = 0.97) and was ~ 7% higher than *ΔV*_*Tr*_ (intercept = 0.07). In the AS subjects, the values *ΔFVC*_*(P-B)*_ were substantially greater for the same range of estimated *ΔV*_*Tr*_ and followed a regression line with an intercept (0.15) twice as high and a slope (1.74) 74% higher than those for the NA subjects. For each subject the difference between the measured *ΔFVC*_*(P-B)*_*/FVC*_*B*_ and the estimated *ΔV*_*Tr*_*/FVC*_*B*_ corresponds to the fraction of the reduction in FVC in addition to that caused by closure of segmental airways. Such fraction was significantly (P<0.05) greater in AS compared with NA subjects.

## Discussion

This study provides imaging evidence that the increase in lung volume during breathing of a subject after bronchoconstriction is not due to passive hyperinflation since it was not positively associated with the average degree of airway constriction by MCh. Rather, the data shows that the change in lung volume was positively associated with, and exceeding by at least 15%, that predicting the onset of the first segmental airway closure. To our knowledge, this is a first study linking inter-subject differences in whole-lung behavior based on measurements of interdependence between individual airways and their surrounding parenchyma.

In previous reports, it was theorized that airway-parenchyma interdependence could limit the degree of airway narrowing and be linked to observed elevations in FRC during bronchoconstriction [6,26]. Some of the early studies, describing an increase in FRC following histamine-induced bronchoconstriction, showed activation of inspiratory muscles during exhalation and thus attributed the effect to active control of breathing [3]. Similarly prolonged post inspiratory electrical activity of the diaphragm and parasternal inter-costal muscles was recorded in bronchoconstricted dogs [27]. However, other studies have attributed the elevation in FRC to dynamic hyperinflation caused by purely passive mechanisms such as the elevation of breathing frequency and/or airway resistance [2,4,28,29] or by gas trapped behind closed airways.

In animal [7,18] and human [30–35] studies, airway distensibility of individual airways by the parenchyma was evaluated with HRCT during elevations in global lung volume. Those studies defined airway distensibility as the percent increase in luminal area measured between images at FRC and TLC with [36–38] or without [34,35,39,40] normalization by the magnitude of the total lung volume change. Although the heterogeneity in airway distensibility appears to be a characteristic of asthma [40], the causes of such heterogeneity and its impact in the whole lung behavior have remained unclear. Among other reasons the following may have contributed to the observed heterogeneity:

1. Distensibility was defined in terms of the changes in airway luminal areas even though the true interface between the airway wall and the peribronchial parenchyma takes place at the outer surface of the airway.
2. The change in airway area and the expansion of the surrounding parenchyma are non-linear functions of global trans-pulmonary pressure, both becoming stiffer at increasing distending pressures [18].
3. The expansion of peribronchial parenchyma may depend not only on the degree of lung inflation, but also on gravitational forces, the shape of the thorax, and the rigidity of airway tree and vascular structures. Thus, normalizing airway distensibility by a global measurement of lung volume would ignore these effects.
4. Expansion of an airway by its surrounding parenchyma depends on the degree of ASM activation. In severely constricted airways, ASM force may exceed all counteracting forces so that a closed airway would remain shut until the sum of expansive forces exceeds that by the ASM.

This paper introduced a novel approach to evaluate experimentally the effects of airway-parenchyma interdependence: 1) A relative distensibility (*RD*) of individual airways was defined in terms of their change in outer cross-sectional area in relation to the expansion of their surrounding parenchyma, and 2) A theoretical framework was presented to link the measured *RD* of individual airways with changes in overall lung volume and vital capacity caused by broncho-constriction.

This approach, applied to the study of broncho-constricted subjects with and without asthma yield the following insights: 1) The expansion of peribronchial parenchyma around an airway and that of the subtended parenchyma were dependent on their relative vertical distance within the lung and the global degree of lung inflation. 2) At baseline, peribronchial expansion around a segmental airway was approximately equal to that of its subtended parenchyma once the effect of the vertical distance between airway and distal parenchyma was accounted for. 3) During broncho-constriction, as lung volume increased, peribronchial parenchyma expanded less than the subtended parenchyma. 4) The change in mean lung volume during breathing following bronchoconstriction was inversely associated with the average degree of airway constriction; a finding inconsistent with passive hyperinflation. 4) Conversely, the relative change in breathing lung volume after bronchoconstriction of a subject was associated with that estimated for closure of his/her first segmental airway. And 5) The relative change in FVC following bronchoconstriction of a subject was proportional, but greater than, the estimated sum of trapped gas behind closing segmental airways during a lung exhalation to RV. These findings and their physiological implications are discussed in more detail below.

### Expansion of the peribronchial and segmental parenchyma

Average peribronchial parenchyma expansion around constricted segmental airways (*E*_*pb*_) was generally different than that of the subtended parenchyma (*E*_*SL*_). Such a difference depended in part on the relative vertical distance between center of gravity of the segmental parenchyma and the peribronchial ROI center (S4 Fig). Such a vertical dependence is consistent with the well known vertical gradient in lung expansion for human lungs in the supine position [41–46]. Indeed, at baseline conditions and once the vertical dependence was accounted for, sub-lobar and peribronchial parenchyma were equally expanded (*E*_*SL*_*- E*_*pb*_~0). However, as lung volume increased after bronchoconstriction, expansion of the peribronchial parenchyma was less than that of the subtended parenchyma, (*E*_*SL*_*- E*_*pb*_>0) with a difference that was correlated with the global degree of lung expansion (*E*_*Lung*_): for either a subject or condition the greater *E*_*Lung*_ the greater (*E*_*SL*_*- E*_*pb*_) (Fig 4). This means that, except for the expected gravitational differences in lung expansion, under normal breathing conditions the parenchyma is uniformly expanded across iso-gravitational planes the lung. However, as the breathing lung volume increased with broncho-constriction, *E*_*pb*_ increased less than *E*_*SL*_ generating the differences observed in parenchymal expansion. A possible cause for this difference could be a local effect of the stiffer airway and vascular trees as predicted by theoretical modeling studies [20] for parenchyma within 4 diameters of the airways.

### Airway-parenchyma interdependence of individual airways and changes in mean lung volume during breathing after MCh challenge

The findings of heterogeneity in parenchymal expansion within the lung may have implications for understanding airway narrowing and for disease diagnosis. Distention of the airways by the parenchyma has been identified as a potent bronchodilator, at least in normal human lungs. Lai-Fook and colleagues [47] evaluated Wilson’s model of airway-parenchyma interdependence [48] and showed that the radial traction exerted by the surrounding parenchyma was the mechanism by which intrapulmonary airways are expanded during a deep inhalation.

The present study evaluated dimensions of individual airways in spontaneously breathing subjects before and after broncho-constriction. By protocol, we allowed the subjects to chose their breathing pattern and mean lung volume both at baseline and after MCh. This not only made the conditions of the study more physiologically relevant, but also allowed us to test whether differences in the change in lung volume among subjects between baseline and post MCh, (*ΔV*_*L*_), could be associated to the average degree of airway constriction (dynamic hyperinflation) or related to the prevention of airway closure by the interdependent behavior between airways and their surrounding parenchyma.

If the elevation of *ΔV*_*L*_ for a subject was merely a passive phenomena of dynamic hyperinflation, one would have expected that *ΔV*_*L*_ */V*_*TLC*_, would have been positively correlated with de average degree of luminal airway narrowing between baseline and post MCh ([*A*_*i*,*B*_—*A*_*i*,*P*_]/*A*_*i*,*B*_). In contrast we found that *ΔV*_*L*_ */V*_*TLC*_ was inversely correlated with [*A*_*i*,*B*_—*A*_*i*,*P*_]/*A*_*i*,*B*_ (Fig 7). Thus, our data is inconsistent with the hypothesis that the elevation of *V*_*L*_ during bronchoconstriction was caused by an increase in airway resistance. Instead, the data showed that the subjects with the greatest degree of luminal narrowing were those that did not increase *V*_*L*_ and, conversely, that subjects with the highest *ΔV*_*L*_ */V*_*TLC*_ had negative values of [*A*_*i*,*B*_—*A*_*i*,*P*_]/ *A*_*i*,*B*_ corresponding to an average segmental airway dilation post MCh.

If the elevation in lung volume post MCh was not associated with passive dynamic hyperinflation, then what could explain it? Our data showed that the values of *ΔV*_*L*_*/V*_*TLC*_ for both AS and NA subjects were inversely associated with the minimal value of *A*_i_*/A_*o*,*T*_ among all segmental airways of each subject (*A*_i_*/A_*o*,*T*_)_min_. Virtually identical results were obtained when the minimal *A*_i_*/A_*o*,*T*_ was calculated as the mean minus two standard deviations of the *A*_i_*/A_*o*,*T*_ distribution for all segmental airway of each subject (S8 Fig). As discussed in the empirical framework, the value of *A*_i_* is a theoretical estimate of the luminal area that the airway would have reached, had the peribronchial parenchyma expansion remained unchanged after MCh, assuming a constant relative distensibility for that constricted airway. In an airway with *A*_i_* = 0 the elevation in smooth muscle force by MCh would be just enough to bring it to closure if peribronchial parenchyma expansion had remained constant after constriction. Similarly, in an airway with *A*_i_*<0 the smooth muscle force would be greater than that by the parenchyma opposing its closure and as a result, if lung volume had not increased after MCh, the airway not only would have become fully closed, but it would have also remained closed until the peribronchial expansion had exceeded the critical value of peribronchial parenchyma expansion (*E*_*pb*,*Crit*_). Our data shows that all subjects had their mean lung volume following bronchoconstriction at levels that were at least 15% greater than those for closure of the first segmental airway on each subject. This remarkable finding could suggest that the behavior of individual airways, and not that of the average airway, may have affected lung volume during breathing after bronchoconstriction. This result derived for airways > = 2mm in diameter, together with previous observations of inspiratory muscle activation during exhalation [3], would be consistent with an active control mechanism aimed at preventing the onset of airway closure during spontaneously breathing, possibly mediated by vagal sensory neurons such as those recently identified in murine airways [49]. However, our findings cannot rule out the presence of other mechanisms. For example air trapping behind airways too small to be measured with CT could have affected lung volume during spontaneous breathing. If that was the case, the behavior of larger airways would have been a marker for that of deeper airways.

### Air trapping and changes in FVC following broncho-constriction

We observed large inter-subject differences in the reduction in FVC during MCh bronchoconstriction (Fig 8). The values of *ΔFVC/FVC*_*B*_ in both groups were associated with, but greater than, the fraction of FVC_B_ expected to become trapped behind closing segmental airways during a forced exhalation from TLC to RV (*ΔV*_*C*_*/FVC*_*B*_). In NA subjects that difference *ΔFVC/FVC*_*B*_ increased in the same proportion as the predicted difference in *ΔV*_*C*_ (slope of the regression line ~1) and was, in average, only slightly higher than *ΔV*_*C*_ (intercept ~ 7%). In the AS subjects, however, the intercept value of *ΔFVC/FVC*_*B*_ was 15% greater than *ΔV*_*C*_*/FVC*_*B*_, for subjects with minimal *ΔV*_*C*_, and increased 74% more than *ΔV*_*C*_*/FVC*_*B*_. These findings show that in subjects with healthy lungs the reduction in FVC during MCH bronchoconstriction is closely related to the amount of gas entrapped behind specific segmental airways. In the asthmatic lungs, however, the estimated gas trapped behind closed segmental airways explains only a fraction of the reduction in FVC. This suggests that in the asthmatic lung a substantial part of the reduction in FVC by MCh may be attributed to gas trapping behind airways more peripheral than the segmental airways.

### Relative airway distensibility, *RD*

*RD* is the ratio between the fractional change in cross-sectional area of an airway and the fractional change in peribronchial parenchymal expansion to the power of ^2/3^ (to make *RD* dimensionless). Airways with *RD* = 1 expand in the same proportion of their surrounding parenchyma not causing any shear distortion of parenchyma. Most of the airways (78%) had *RD* ≤ 1 and thus expanded less than the surrounding parenchyma, thus resulting in shear distortion of the parenchyma during lung expansion. The rest of the airways had values of *RD* > 1 and thus expanded more than the surrounding parenchyma. This can only take place when the airway wall tissue is very compliant, which is unexpected for segmental airways, or when the peribronchial parenchyma around a severely constricted airway generates forces greater than those of by ASM, resulting in brisk airway dilation. This behavior has been described as fluidizing of a frozen smooth muscle by expanding forces [50]. In average *RD* was less than unity and not different between AS and NA subjects (S6 Fig). This means that MCh-constricted segmental airways in average distend less than the surrounding parenchyma during a deep inhalation to TLC and that airway distensibility does not differentiate mild-to-moderate asthmatic from healthy lungs. This finding is consistent with that from Brown et al [34] and Williamson et al [51] and thus supports the concept that the lack of a bronchodilatory effect of deep inhalations may not be related to a difference in distensibility of the airways or the parenchyma, at least up to the level of segmental airways imaged in this study. In contrast, other studies assessing global airway distensibility indirectly have shown reduced distensibility in subjects with asthma [30,52]. The cause for this apparent disparity in results may be that indirect measurements of distensibility include a contribution from the small airways (less than 2mm^2^ in area), which are not measured using HRCT. It is also possible that the ASM of asthmatics may recover differently from a stretch than NA [34]. However it is unlikely that this disparity will be resolved until the direct measurement of the distensibility of much smaller airways becomes feasible.

In both subjects with asthma and healthy controls values of *RD* varied between -1 (minimum) and 3 (maximum). When airways have distensibility different from unity, they expand differently than peribronchial parenchyma when lung volume is changed, thus generating increased shear stresses on the parenchyma in the vicinity of the airway [48]. When *RD* < 1, the airway is relatively stiffer than the surrounding parenchyma. This may occur when parenchymal tethering forces are unable to overcome the airway smooth muscle forces or when the airway is already near full expansion at MLV. This past behavior is consistent with the reduction in airway lumen distensibility following albuterol observed in AS subjects [30]. Although a few airway with *RD* < 0 were observed, these were airways that their outer area, measured at MLV after MCh, was higher than that measured at TLC. Because most of these airways had minimally changed their area after MCh, this could be caused by imaging or segmentation errors but they could also reflect and unlikely effect of airway constriction in response to the lung expansion to TLC.

The *RD* of individual airways was found to be highly heterogeneous within and between subjects. Previous studies measuring the distensibility of individual airways with HRCT [33,53], recognized they could be potentially important as they were affected by differences in trans-pulmonary pressure, and their transmission within the lungs via the fibrous and elastic stroma, and modified by disease processes [26]. Thus those measures of airway distensibility could be affected by heterogeneity in peribronchial parenchyma expansion amongst airways and by differences in the degrees of lung inflation at TCL and FRC among subjects. Past studies have attempted to control the lung volume during imaging at FRC [36–38] to minimize these effects. However, even if the accurate control of lung volume was possible, regional difference in parenchymal expansion within the lung could still contribute to the heterogeneity in airway distensibility observed.

### Methodological considerations and study limitations

Other methodological differences in our measurements of distensibility compared with those by others are: 1) Airway dimensions and lung expansion were referenced to those measured at TLC instead the less reproducible FRC [54]. 2) We used outer airway cross-sectional area (*A*_*o*_) instead of luminal area (or inner diameter) because it is the dimension directly affecting and/or being affected by the surrounding parenchyma. 3) Airway cross-sectional areas were measured objectively by the PW2 software package, as the average across the middle third of their respective length. This allowed us to assess most segmental airways in all subjects objectively in three conditions *B*, *P*, and *T*. Early measures of airway %distensibility were limited to airways running perpendicular to the CT cross-sections that could have been affected by longitudinal heterogeneity of airway luminal area [19]. Estimation of %distensibility of bronchoconstricted airways (that is between *A*_*i*,*P*_ and *A*_*i*,*T*_) gave average values and heterogeneities consistent with those previously reported for studies in asthmatics post MCh [31,55].

Other methodological details are worth mentioning. Parenchymal Expansion, *E*, was defined as the average gas-to-tissue volumes ratio within the region and was calculated for individual ROI’s from the CT scans. Taking the tissue volume as a surrogate of the number of alveolar units within the ROI, *E* would represent the average degree of gas inflation per alveolar unit.

The resolution of the HRCT scanner could have affected the results. However, airway area measurements using Pulmonary Workstation 2.0 have been validated using phantom tubes with a cross-sectional area greater than 3.1mm^2^ for all scan directions [23] and all airways analyzed within the current study had lumen areas greater or equal to 4.2 mm^2^. Also, partial volume effects could have resulted in underestimation of parenchymal tissue expansion in peribronchial ROI’s because of their smaller dimensions compared with those of the distal segmental ROI’s. However, this is unlikely because the value of *ΔE* increased with lung inflation and the potential partial volume effects should have been greatest at baseline mean lung volume where airway dimensions were the lowest. Nonetheless, it was precisely at low lung expansion where that peribronchial parenchymal expansion approached that of the distal regions (i.e. *ΔE* ~ 0).

We imaged the airways at each subject’s spontaneous MLV both before and after the MCh challenge allowing the evaluation of *A*_*o*_ during physiological conditions. This also removed the need to enforce a breathing pattern and breath-holds at volumes lower than that chosen by the subject after the administration of MCh. Indeed, analysis of the CT scans showed most subjects in both groups increased their MLV following MCh, an outcome that has an independent influence on airway cross-sectional area. In order to account for that effect, based on the relative distensibility of each airway, we estimated the value of (*A*_o_*) as the *A*_*o*_ that would have been measured, had the peribronchial expansion remained unchanged after the MCh challenge. *RD* was calculated assuming that relationship between *A*_*o*_*/A*_*o*,*T*_ and the relative changes in peribronchial expansion (to the ^2/3^ power) was linear. This seems to be a reasonable assumption, for bronchoconstricted airways, based on the measurements by Brown et al. in dogs after aerosol challenge [18], as shown in S7 Fig. Although the linearity assumption is only an approximation, as mentioned above, the impressive correlation between the behavior of specific airways and the response of the system as a whole suggests that such assumption may be a reasonable for broncho-constricted airways.

### Measuring *E* and defining parenchymal ROI’s

Our definition of *E* is similar to the specific gas volume (*SV*_*g*_) [56,57] defined as volume of gas per gram of tissue (ml/g) but it is dimensionless. The quantity of average voxel gas-to-tissue volume ratio (average *E*_*voxel*_), given for lung (*E*_*Lung*_), segment (*E*_*SL*_) and peribronchial region (*E*_*pb*_), represents a measure of parenchymal distension, if one assumes that all alveolar units have the same mass and that it does not change with lung inflation. Clearly changes in blood volume within parenchymal ROI’s could affect the results. We restricted the peribronchial ROI size to be a spherical region with a diameter equal to four times the outer diameter of the airway based on models of parenchyma deformation and airway-airway interaction [25,48]. Given that the outer diameter of the airways changed with the different conditions, we defined the peribronchial ROI at baseline and iteratively adjusted the diameter of the peribronchial ROI for other conditions to include the same amount of tissue than at baseline. However, we repeated the analysis defining the peribronchial ROI’s with a diameter equal to 4 times the outer airway diameter for each condition and that did not change the findings or conclusions of our study.

### Physiological and clinical implications

In a heterogeneously expanding lung a regional reduction in lung expansion decreases parenchymal recoil [58] reduce stretching forces on the airway wall and increase the constriction created by the airway smooth muscle [59]. This can potentiate a positive feedback mechanism promoting further decrease in airway lumen and airflow until the airway becomes unstable and closes [58], contributing to the formation of ventilation defects or VDef’s [44]. The redistribution of airflow to regions away from VDef’s increases their tidal expansion, dilating and stabilizing the corresponding branches of the airway tree. During bronchoconstriction these competing effects result in a bi-stable behavior of airway lumen that leads to the self-organized formation of VDef’s [9]. The heterogeneous behavior of *RD* and A* amongst a subject’s airways is consistent with the above model.

In practical terms, assessing individual airway *RD* and A* could be important in diagnostics, assessment of disease progression and response to therapy, as well as pre-interventional planning [60,61]. HRCT can allow assessment of the contributions of airway and parenchymal changes, and evaluate how these measures relate to symptoms and to pulmonary function [26]. Although our study did not include patients with COPD, we could extrapolate them to this group of patients where the loss of airway attachments and elastic parenchyma recoil result in dynamic collapse of airways during expiration. Wille and Petersen [62] observed a markedly reduced global distensibility of airways in patients with severe COPD. Because our measurement of *RD* includes the effect of distending forces of the surrounding parenchyma, we speculate that this measurement could provide a more sensitive indicator of the disease than that of absolute distensibility. Another potential application of our methodology could be for personalized treatment of severe asthmatics by selecting airways for bronchial thermoplasty treatment [60] based on their values of *RD* or *A*_i_*/A_*o*,*T*_.

## Summary and conclusions

The main findings of our study are consistent with the concept that the elevation of lung volume following bronchoconstriction is not passive hyperinflation but rather an actively controlled process aimed at keeping open the least stable segmental airway of the lung. If true, this mechanistic insight may lead to improved understanding of asthma symptoms and their management. Furthermore, the concept of relative distensibility and the methods presented in this paper could also have clinical applications in early diagnosis and/or treatment of other diseases affecting airways and parenchyma.

## Supporting information

S1 Fig Segmentation of the peribronchial ROI illustrated for airway LB4 of an AS patient. Views are axial (A), sagital (B), coronal (C), and a 3D rendering of both airway and ROI (D). Cavities on the ROI represent non-parenchymal tissue (i.e. bronchial tissue, pulmonary vessels, lymphatic tissues, and nerves).
(PDF)

S2 Fig Examples of voxel gas-to-tissue volume ratio (*E*) Histograms, within the peribronchial ROI (dark colors) and the corresponding distal segmental ROI (light colors) fed by the same segmental bronchus. The three plots illustrate the changes in parenchymal expansion linked to a RB9 bronchus of a representative AS subject measured at MLV during baseline (*B*, upper and green), post Methacholine challenge (*P*, middle and red) and at TLC post challenge (*P*, lower and blue). The example was chosen because the height difference between geometric centers of the peribronchial and distal segmental ROI’s were close to zero similar for the three conditions.
(PDF)

S3 FigExample of average expansion ($\overset{-}{E}$) of the parenchyma in a peribronchial region of interest (Epb, solid circles) and in the segmental distal parenchyma it feeds (ESL, open circles), in the three conditions studied: baseline at baseline (B, green), post MCh challenge (P, red), and post challenge at TLC (P, blue).The data is from the airway RB8 of an AS subject. Note that, in this case the peribronchial expansion is always greater than that of the corresponding distal parenchyma, and the gradient in expansion (*ΔE*) increases from B to P to T even though the center of the peribronchial sphere was at approximately at the same vertical level of the geometric center of the subtended segmental ROI.(PDF)

S4 Fig Gradient in expansion between segmental distal parenchyma and the corresponding peribronchial parenchyma (ΔE) as a function of the relative-vertical-distance between the center point of each segmental airway and geometric center of the segmental parenchyma it feeds, measured at MLV at baseline (B, green), post MCh challenge (P, red) and at TLC post challenge (P, blue) The two plots illustrate the relationships for an subject with asthma (top) and one without asthma (bottom).
(PDF)

S5 Fig Values of *A_i,P_/A_o,T_* vs. *A*_i_*/A_*o*,*T*_ for all individual airways of all subjects studied (open symbols for subjects without asthma (NA) and closed symbols for subjects with AS). The airway of subjects with the most negative value of *A*_i_*/A_*o*,*T*_ are marked with arrows. Also note that that several subjects (AS6, AS3, AS7, NA3 and NA8) had more than one airway with negative values ***A***_i_*/A_***o*,*T***_ corresponding to airways that would have closed had the subjects not increased their lung volume after MCh challenge.
(PDF)

S6 Fig Individual airway values of RD for each of the 19 sublobar regions (subtended by each airway), for all subjects studied. *RD* of individual airways was highly heterogeneous between airways and among subjects. *RD* varied from close to zero in some airways to as high as three in others including regions with values above and below unity.
(PDF)

S7 FigComparison between average results from this study for subjects with asthma (black) and subjects without asthma (red) and experimental animal data [18] (blue).For the human data (mean +/- SEM for all segmental airways of all subjects of each group) the plots present the relationship between, bronchial luminal area A_i_ elevated to the ^3/2^ power as a percentage of the equivalent area at TLC (*A*_*i*,*T*_)^2/3^, as a function of peribronchial parenchymal expansion *E*_*pb*_ as a percentage of *E*_*pb*_ at TLC (*E*_*pb*,*T*_) at three conditions: baseline (*B*), post MCh (*P*) and TLC (*T*). The value of *A*_*i*_ that would have been expected had there been no change *E*_*pb*_ between B and P ($A_{i}^{*}$) was estimated by extrapolation assuming that the slope between the points P and T for each airway was the same as between P and P*. To present the animal data in equivalent units *E*_*PB*_ is estimated from reported lung volume values for six anesthetized dogs measured at five airway pressures (0, 12, 25, 32, and 45 cm H_2_O) under two conditions: baseline (open blue squares), and after a strong aerosol histamine challenge (200 mg/ml, 5 breaths to 15 cm H_2_O, solid blue squares.(PDF)

S8 Fig Comparison of two methods for calculating the (*A*_i_*/A_*o*,*T*_)*min*. 1). The minimum value of *A*_i_*/A_*o*,*T*_ for each subject (X axis) and 2) defined as the mean minus two standard deviation of the *A*_i_*/A_*o*,*T*_ distribution for each subject. Each data point corresponds to a subject of the AS (blue) and NA (red) groups. Note that both methods follow closely the identity line.
(PDF)

## Abbreviations

*ΔE*: Difference in parenchymal expansion between segmental and peribronchial parenchyma ROIs
*ΔE_mean_*: Average difference between segmental and peribronchial expansion after accounting for the vertical location dependence
*Δh*: Relative vertical-distance between the airway’s center point and the corresponding geometric center of the subtended segmental parenchymal ROI normalized by lung maximal dorso-ventral height
*ΔV_C_*: Estimated critical (minimum) change in lung volume at the onset of the first airway closure
*A**: Theoretical estimate of cross-sectional (inner, *i*; or outer, *o*) area that the airway would have reached, had the peribronchial lung expansion remained unchanged after MCh
*A_i_*: Inner airway cross-sectional area
*A_o_*: Outer airway cross-sectional area
*A_w_*: Airway wall area
*B*: Baseline
*E_Lung_*: Average lung parenchyma expansion
*E_pb_*: Peribronchial parenchyma expansion
*E_pbCrit_*: Critical peribronchial expansion
*E_SL_*: Parenchyma expansion of the subtended lung segment
*P*: Post metacholine (MCh)
*RD*: Relative Distensibility of the airway in relation to that of the surrounding parenchyma
*T*: Total lung capacity
*V_Tr_*: Estimated trapped air volume
