# Supplementary material for: Elevation in lung volume and preventing catastrophic airway closure in asthmatics during bronchoconstriction
Source: PLoS One. 2018 Dec 19;13(12):e0208337. doi: 10.1371/journal.pone.0208337 (PMC6300269; doi:10.1371/journal.pone.0208337)
Supplement: S1 Fig — Views are axial (A), sagital (B), coronal (C), and a 3D rendering of both airway and ROI (D). Cavities on the ROI represent non-parenchymal tissue (i.e. bronchial tissue, pulmonary vessels, lymphatic tissues, and nerves). (PDF) [file pone.0208337.s001.pdf]

Visualization of the peribronchial parenchymal ROI's typically showed cavities within the ROI's representing the exclusion of vessels and airway wall (S1 Fig).

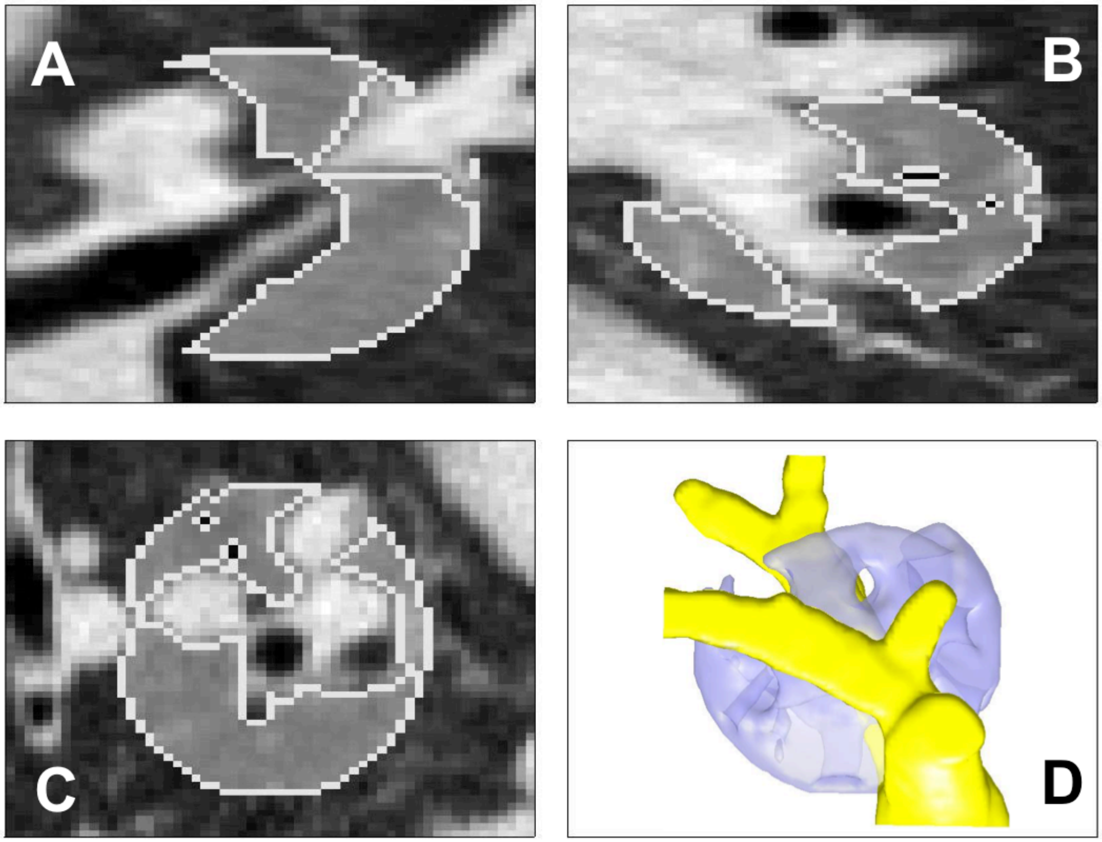

S1 Fig. Segmentation of the peribronchial ROI illustrated for airway LB4 of an AS patient. Views are axial (A), sagital (B), coronal (C), and a 3D rendering of both airway and ROI (D). Cavities on the ROI represent non-parenchymal tissue (i.e. bronchial tissue, pulmonary vessels, lymphatic tissues, and nerves).

The process for obtaining the parenchymal masks described in the methods should be further discussed. The additional elimination of airway wall tissue, vessels, and nerves, using the threshold of -950 to -400 Hounsfield units (HU), was justified as follows: Mullan et al. used a cut-off of 40% air content (corresponding to -361HU) to limit effects of volume averaging from blood vessels with adjacent lung [17], while Aliverti et al. used a similar

high threshold value (-400HU) for excluding blood vessels [14]. The lower threshold value of (-950HU) has been used for excluding hyper-inflated tissue [15,16]. The resulting mask mainly includes parenchyma with normal aerated tissue [13], which is reasonable since we do not expect our study subjects have emphysema. However, recognizing that the selection of threshold had could have an important effect on the effects being studied, we conducted additional analysis testing the effect of varying the threshold values and found that when within plausible ranges the main results and conclusions from the study were not affected. We also recognize that the size of the spherical ROI for peribronchial parenchyma analysis could potentially affect the results since at different degrees of lung inflation could be sampling different peribronchial tissue. For that reason, we implemented an iterative algorithm to keep the tissue volume of the ROI at *P* and *T* to be within 5% of that of the ROI at *B*, so that approximately the same tissue was included in the 3 ROI's. Finally, partial volume effects could also affect the calculations peribronchial and distal lung expansion, especially at *B* where the mean HU value of the parenchyma is closer to the HU value of the airway wall (less inflation). This potential error, however, would have cause an increase in the difference between peribronchial and subtended parenchymal expansion at *B*, but the results showed that this difference was actually close to zero at this condition.
