# Supplementary material for: Elevation in lung volume and preventing catastrophic airway closure in asthmatics during bronchoconstriction
Source: PLoS One. 2018 Dec 19;13(12):e0208337. doi: 10.1371/journal.pone.0208337 (PMC6300269; doi:10.1371/journal.pone.0208337)
Supplement: S2 Fig — The three plots illustrate the changes in parenchymal expansion linked to a RB9 bronchus of a representative AS subject measured at MLV during baseline (B, upper and green), post Methacholine challenge (P, middle and red) and at TLC post challenge (P, lower and blue). The example was chosen because the height difference between geometric centers of the peribronchial and distal segmental ROI’s were close to zero similar for the three conditions. (PDF) [file pone.0208337.s002.pdf]

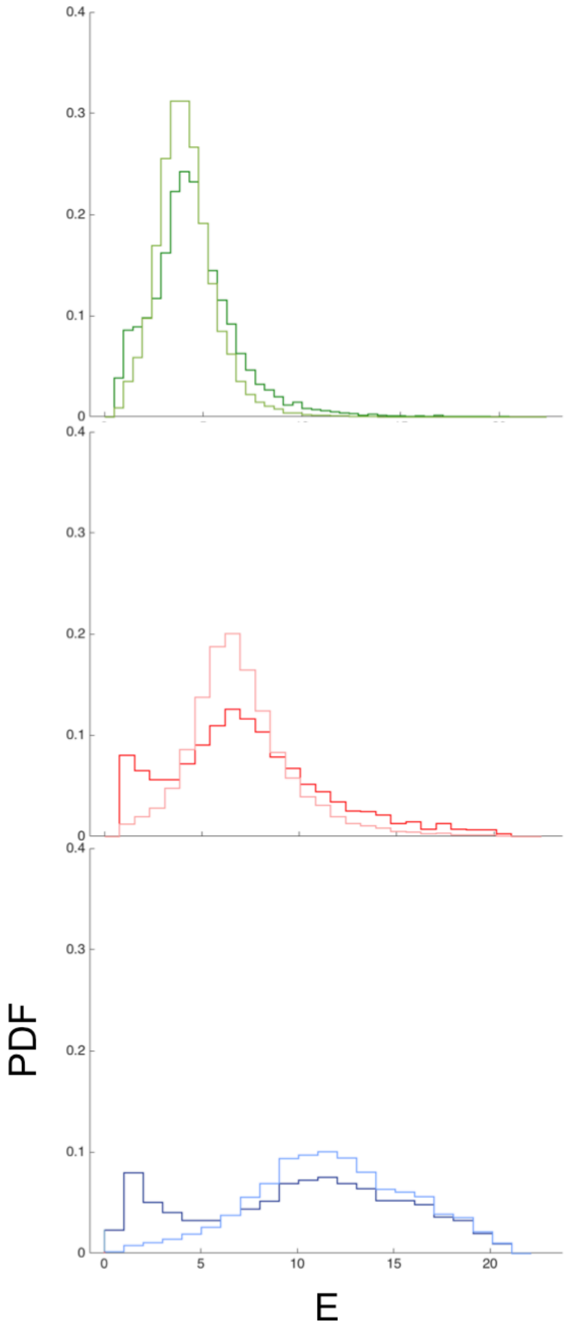

S2 Fig. Examples of voxel gas-to-tissue volume ratio (*E*) Histograms, within the peribronchial ROI (dark colors) and the corresponding distal segmental ROI (light colors) fed by the same segmental bronchus. The three plots illustrate the changes in parenchymal expansion linked to a RB9 bronchus of a representative AS subject measured at MLV during baseline (*B*, upper and green), post Methacholine challenge (*P*, middle and red) and at TLC

post challenge (*P*, lower and blue). The example was chosen because the height difference between geometric centers of the peribronchial and distal segmental ROI's were close to zero similar for the three conditions.
