# Supplementary material for: Elevation in lung volume and preventing catastrophic airway closure in asthmatics during bronchoconstriction
Source: PLoS One. 2018 Dec 19;13(12):e0208337. doi: 10.1371/journal.pone.0208337 (PMC6300269; doi:10.1371/journal.pone.0208337)
Supplement: S3 Fig — The data is from the airway RB8 of an AS subject. Note that, in this case the peribronchial expansion is always greater than that of the corresponding distal parenchyma, and the gradient in expansion (ΔE) increases from B to P to T even though the center of the peribronchial sphere was at approximately at the same vertical level of the geometric center of the subtended segmental ROI. (PDF) [file pone.0208337.s003.pdf]

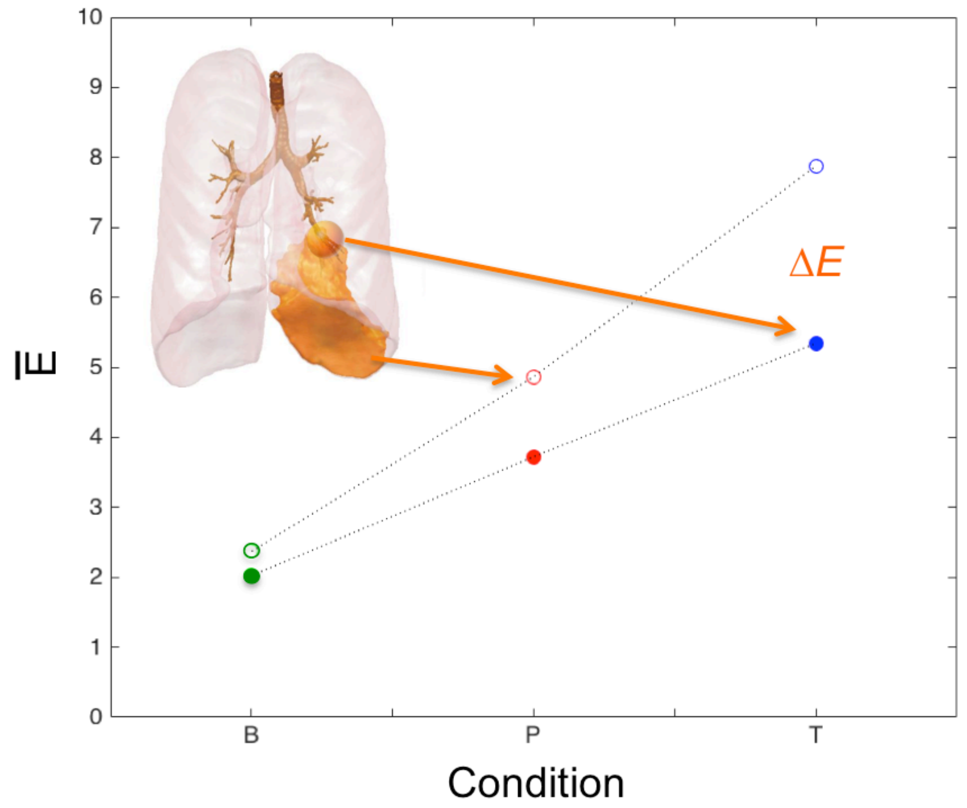

S3 Fig. Example of average expansion ($\bar{E}$) of the parenchyma in a peribronchial region of interest (E_{pb}, solid circles) and in the segmental distal parenchyma it feeds (E_{SL}, open circles), in the three conditions studied: baseline at baseline (*B,* green), post MCh challenge (*P,* red), and post challenge at TLC (*P,* blue). The data is from the airway RB8 of an AS subject. Note that, in this case the peribronchial expansion is always greater than that of the corresponding distal parenchyma, and the gradient in expansion (ΔE) increases from *B* to *P* to *T* even though the center of the peribronchial sphere was at approximately at the same vertical level of the geometric center of the subtended segmental ROI.
