# Supplementary material for: Elevation in lung volume and preventing catastrophic airway closure in asthmatics during bronchoconstriction
Source: PLoS One. 2018 Dec 19;13(12):e0208337. doi: 10.1371/journal.pone.0208337 (PMC6300269; doi:10.1371/journal.pone.0208337)
Supplement: S4 Fig — The two plots illustrate the relationships for an subject with asthma (top) and one without asthma (bottom). (PDF) [file pone.0208337.s004.pdf]

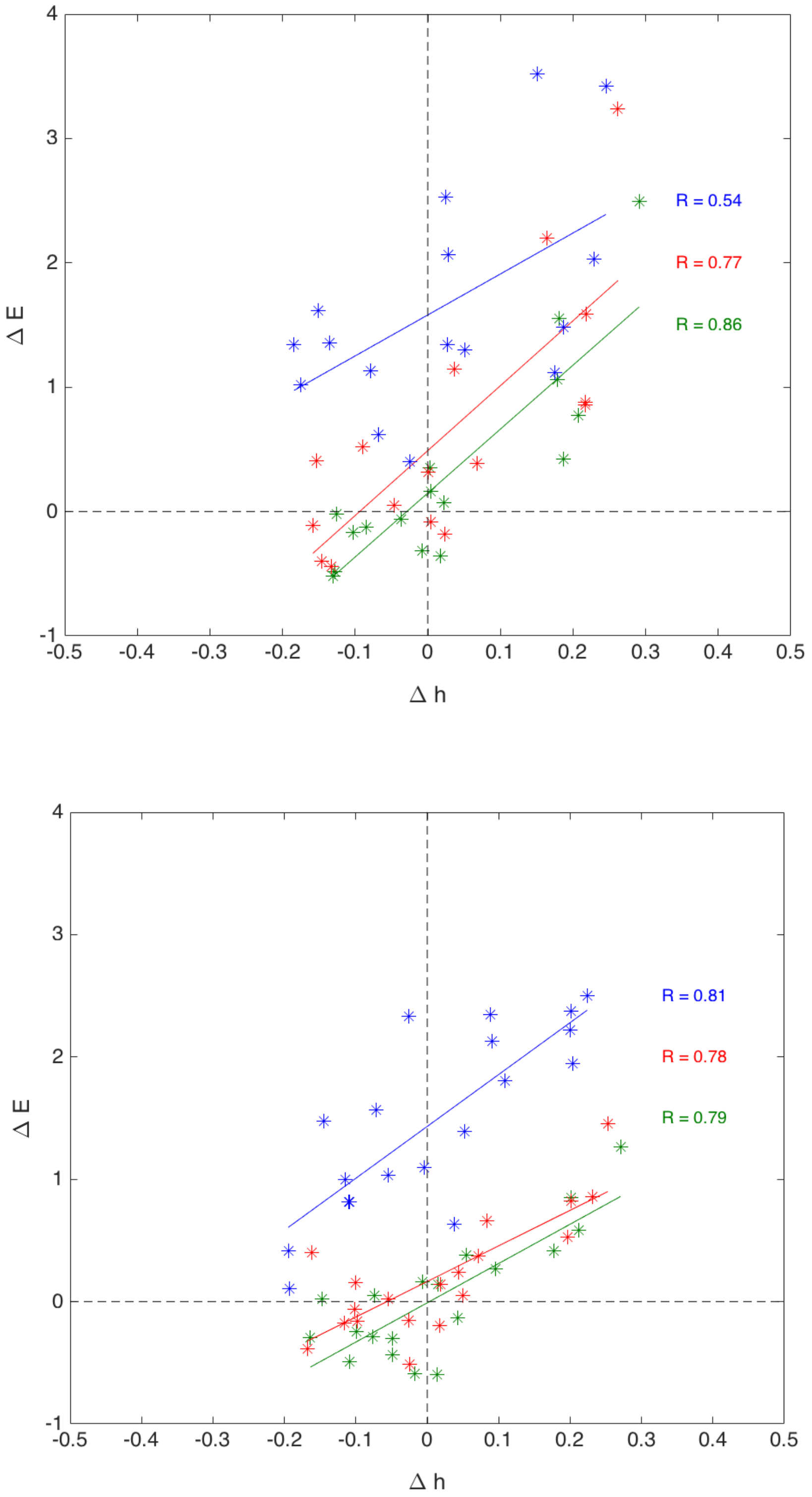

S4 Fig. Gradient in expansion between segmental distal parenchyma and the corresponding peribronchial parenchyma (*ΔE)* as a function of the relative-

vertical-distance between the center point of each segmental airway and geometric center of the segmental parenchyma it feeds, measured at MLV at baseline (*B*, green), post MCh challenge (*P*, red) and at TLC post challenge (*P*, blue) The two plots illustrate the relationships for an subject with asthma (top) and one without asthma (bottom).

The strength of the vertical dependency (R^2 value) was significantly higher in the NA group compared with the AS group for both *P* and *T* conditions (P<0.05). This was also the case for the slope of the regression line with relative height among the different segmental airways in the *T* condition that was significantly higher in NA (3.68 +/-1.11) compared to AS (2.69 +/-0.83, P<0.05, Table 2).
