# Supplementary material for: Elevation in lung volume and preventing catastrophic airway closure in asthmatics during bronchoconstriction
Source: PLoS One. 2018 Dec 19;13(12):e0208337. doi: 10.1371/journal.pone.0208337 (PMC6300269; doi:10.1371/journal.pone.0208337)
Supplement: S5 Fig — The airway of subjects with the most negative value of Ai*/Ao,T are marked with arrows. Also note that that several subjects (AS6, AS3, AS7, NA3 and NA8) had more than one airway with negative values Ai*/Ao,T corresponding to airways that would have closed had the subjects not increased their lung volume after MCh challenge. (PDF) [file pone.0208337.s005.pdf]

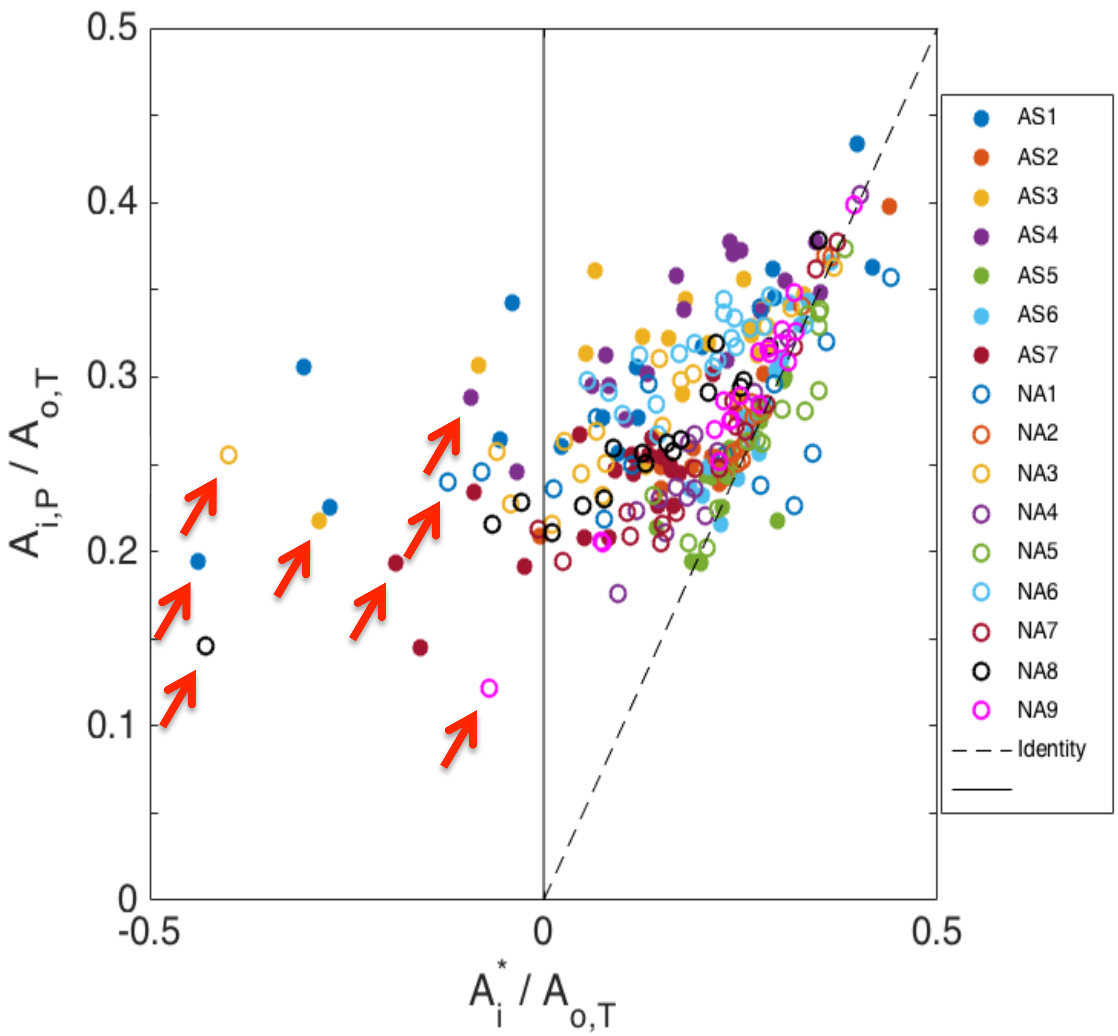

S5 Fig. Values of $A_{i,P}/A_{o,T}$ vs. $A_i*/A_{o,T}$ for all individual airways of all subjects studied (open symbols for subjects without asthma (NA) and closed symbols for subjects with AS) The airway of subjects with the most negative value of $A_i*/A_{o,T}$ are marked with arrows. Also note that that several subjects (AS6, AS3, AS7, NA3 and NA8) had more than one airway with negative values $A_i*/A_{o,T}$ corresponding to airways that would have closed had the subjects not increased their lung volume after MCh challenge.
