# Supplementary material for: Elevation in lung volume and preventing catastrophic airway closure in asthmatics during bronchoconstriction
Source: PLoS One. 2018 Dec 19;13(12):e0208337. doi: 10.1371/journal.pone.0208337 (PMC6300269; doi:10.1371/journal.pone.0208337)
Supplement: S6 Fig — RD of individual airways was highly heterogeneous between airways and among subjects. RD varied from close to zero in some airways to as high as three in others including regions with values above and below unity. (PDF) [file pone.0208337.s006.pdf]

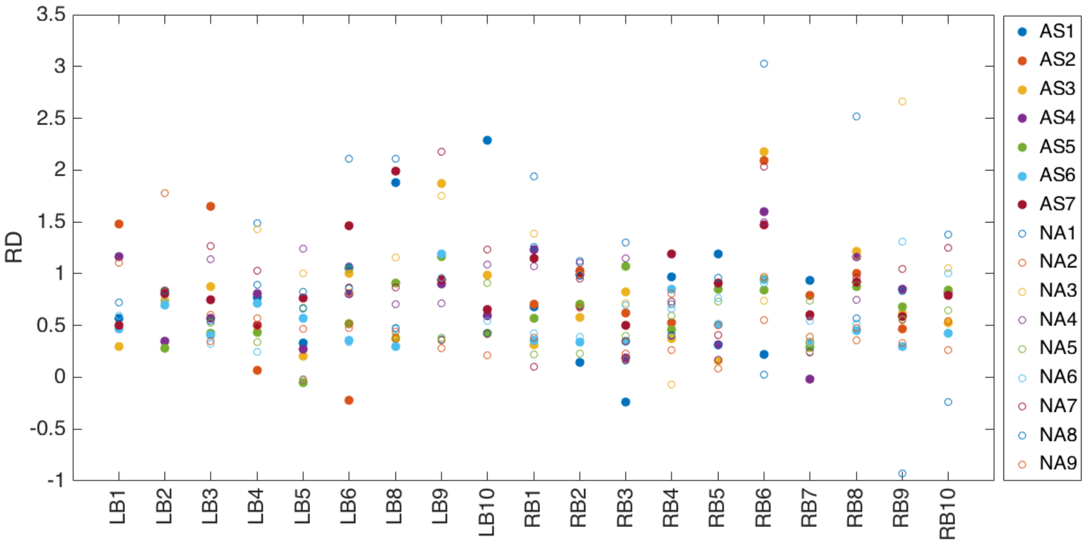

S6 Fig. Individual airway values of *RD* for each of the 19 sublobar regions (subtended by each airway), for all subjects studied. *RD* of individual airways was highly heterogeneous between airways and among subjects. *RD* varied from close to zero in some airways to as high as three in others including regions with values above and below unity.
