# Supplementary material for: Elevation in lung volume and preventing catastrophic airway closure in asthmatics during bronchoconstriction
Source: PLoS One. 2018 Dec 19;13(12):e0208337. doi: 10.1371/journal.pone.0208337 (PMC6300269; doi:10.1371/journal.pone.0208337)
Supplement: S7 Fig — For the human data (mean +/- SEM for all segmental airways of all subjects of each group) the plots present the relationship between, bronchial luminal area Ai elevated to the 3/2 power as a percentage of the equivalent area at TLC (Ai,T)2/3, as a function of peribronchial parenchymal expansion Epb as a percentage of Epb at TLC (Epb,T) at three conditions: baseline (B), post MCh (P) and TLC (T). The value of Ai that would have been expected had there been no change Epb between B and P (Ai*) was estimated by extrapolation assuming that the slope between the points P and T for each airway was the same as between P and P*. To present the animal data in equivalent units EPB is estimated from reported lung volume values for six anesthetized dogs measured at five airway pressures (0, 12, 25, 32, and 45 cm H2O) under two conditions: baseline (open blue squares), and after a strong aerosol histamine challenge (200 mg/ml, 5 breaths to 15 cm H2O, solid blue squares. (PDF) [file pone.0208337.s007.pdf]

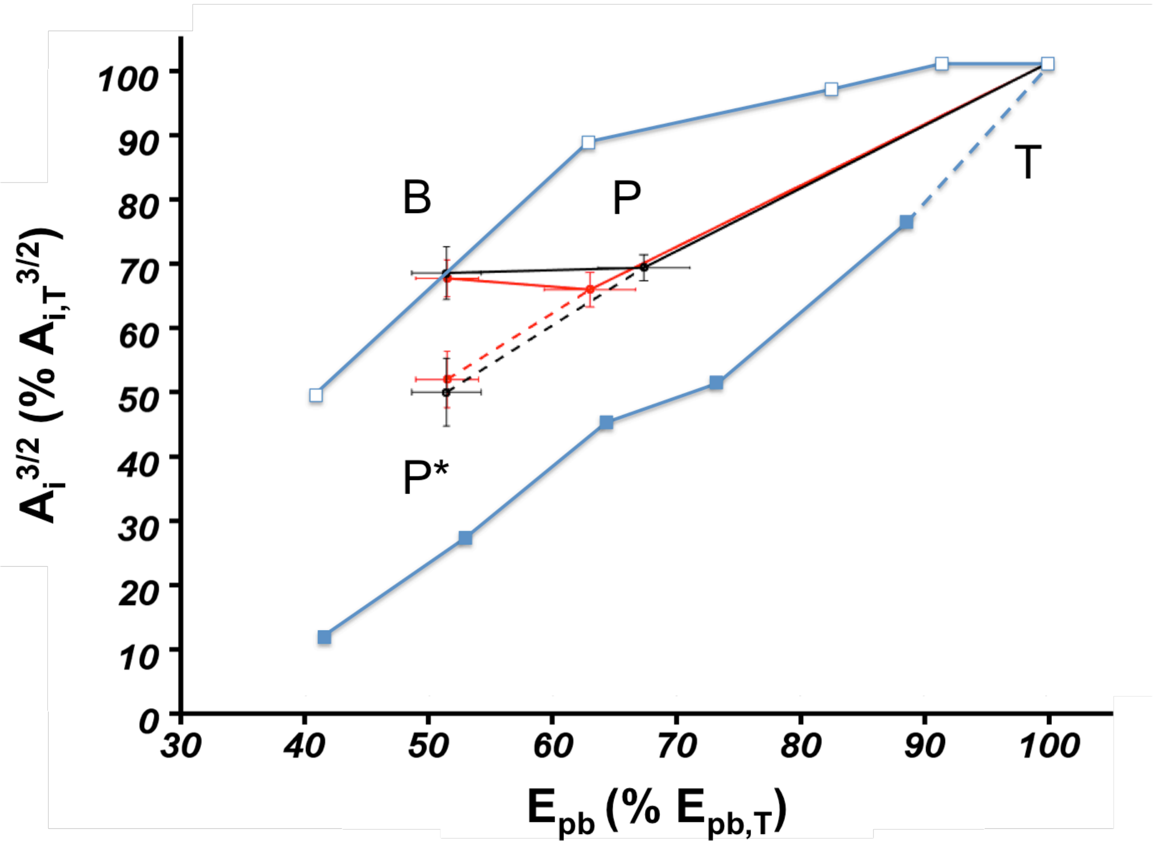

S7 Fig. Comparison between average results from this study for subjects with asthma (black) and subjects without asthma (red) and experimental animal data [13] (blue) For the human data **(**mean +/- SEM for all segmental airways of all subjects of each group) the plots present the relationship between, bronchial luminal area A_i elevated to the $^{3/2}$ power as a percentage of the equivalent area at TLC $(A_{i,T})^{2/3}$, as a function of peribronchial parenchymal expansion E_{pb} as a percentage of E_{pb} at TLC ($E_{pb,T}$) at three conditions: baseline (B), post MCh (P) and TLC (T). The value of A_i that would have been expected had there been no change E_{pb} between B and P (A_i^*) was estimated by extrapolation assuming that the slope between the points P and T for each airway was the same as between P and P*.

To present the animal data in equivalent units E_{PB} is estimated from reported lung volume values for six anesthetized dogs measured at five airway pressures (0, 12,

25, 32, and 45 cm H_2O) under two conditions: baseline (open blue squares), and after a strong aerosol histamine challenge (200 mg/ml, 5 breaths to 15 cm H_2O, solid blue squares).
