# Supplementary material for: Elevation in lung volume and preventing catastrophic airway closure in asthmatics during bronchoconstriction
Source: PLoS One. 2018 Dec 19;13(12):e0208337. doi: 10.1371/journal.pone.0208337 (PMC6300269; doi:10.1371/journal.pone.0208337)
Supplement: S8 Fig — 1). The minimum value of Ai*/Ao,T for each subject (X axis) and 2) defined as the mean minus two standard deviation of the Ai*/Ao,T distribution for each subject. Each data point corresponds to a subject of the AS (blue) and NA (red) groups. Note that both methods follow closely the identity line. (PDF) [file pone.0208337.s008.pdf]

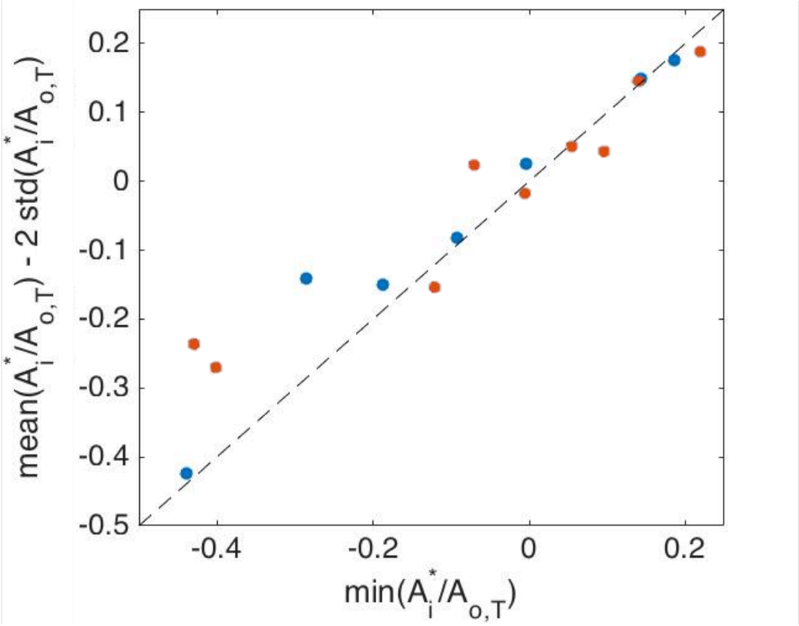

S8 Fig. Comparison of two methods for calculating the *($A_i^*/A_{o,T}$)min*. 1) the minimum value of $A_i*/A_{o,T}$ for each subject (X axis) and 2) defined as the mean minus two standard deviation of the $A_i*/A_{o,T}$ distribution for each subject. Each data point corresponds to a subject of the AS (blue) and NA (red) groups. Note that both methods follow closely the identity line.
